# Integrated In Vitro, In Vivo, and In Silico Evaluation of Antioxidant, Anti-Inflammatory, Analgesic, and Anti-Arthritic Activities of Selected Marine Species

**DOI:** 10.3390/bioengineering13020158

**Published:** 2026-01-28

**Authors:** Md. Jahin Khandakar, Ainun Nahar, Md. Wahidul Alam, Md. Jahirul Islam Mamun, Abu Sayeed Muhammad Sharif, Asef Raj, Md. Enamul Hoque, Israt Sultana Isha, Nafisa Nawsheen, Saika Ahmed, Md Riasat Hasan, Abu Bin Ihsan, Takashi Saito

**Affiliations:** 1 Department of Oceanography, Faculty of Marine Sciences and Fisheries, University of Chittagong, Chittagong 4331, Bangladesh; khandakarjahin@gmail.com (M.J.K.); wahidul.alam@cu.ac.bd (M.W.A.); enamul_imsf@cu.ac.bd (M.E.H.); isratisha055@gmail.com (I.S.I.); 2 Department of Pharmacy, School of Life Sciences, Eastern University, Dhaka 1345, Bangladesh; tahira.ainun@gmail.com; 3 Department of Pharmacy, Faculty of Biological Sciences, University of Chittagong, Chittagong 4331, Bangladesh; jahirulmamun3@gmail.com (M.J.I.M.); nafisanawsheen333@gmail.com (N.N.); 4 Department of Biological Oceanography, Bangladesh Oceanographic Research Institute, Cox’s Bazar, Chittagong 4750, Bangladesh; sharif.asm@bori.gov.bd; 5 School of Pharmacy, BRAC University, Dhaka 1212, Bangladesh; raj.asef@gmail.com; 6 Department of Chemistry, University of Dhaka, Dhaka 1000, Bangladesh; saika@du.ac.bd; 7 Division of Clinical Cariology and Endodontology, Department of Oral Rehabilitation, School of Dentistry, Health Sciences University of Hokkaido, Tobetsu 061-0293, Hokkaido, Japan; riasat@hoku-iryo-u.ac.jp

**Keywords:** marine drug, seaweed, phytochemicals, antioxidant, analgesic, antiarthritic, anti-inflammatory

## Abstract

Marine ecosystems represent a largely untapped reservoir of bioactive compounds with significant pharmacological potential. This study aimed to evaluate the therapeutic properties of ethanol extracts from four marine species: *Padina australis*, *Spatoglossum asperum*, *Holothuria (Halodeima) atra*, and *Hypnea valentiae*. Phytochemical screening, along with a comprehensive series of in vitro, in vivo, and in silico assays, was performed to evaluate the extracts’ pharmacological activities, including antioxidant potential (2,2-diphenyl-1-picrylhydrazyl assay), anti-inflammatory effect (carrageenan-induced paw edema method), analgesic activity (acetic acid-induced writhing and tail immersion tests), and anti-arthritic efficacy (protein denaturation assay). The extracts were found to be rich in flavonoids, tannins, alkaloids, saponins, glycosides, and phenolic compounds, which may underlie the observed bioactivities. In the acetic acid–induced writhing test, *Hypnea valentiae* at 400 mg/kg exhibited the highest peripheral analgesic activity, producing 82.51% inhibition of writhing (*p* < 0.001). In the tail immersion assay, *Padina australis* at doses of 200 and 400 mg/kg showed significant central analgesic effects, as evidenced by increased latency time and percentage of maximum possible effect (MPE). In the carrageenan-induced paw edema model, several treatment groups, including *Padina australis*, *Hypnea valentiae*, *Spatoglossum asperum*, and *Holothuria atra*, at both tested doses showed marked suppression of inflammation, with some groups achieving complete inhibition (100%; *p* < 0.001) at 120 min. The ethanol extract of *Holothuria atra* exhibited the strongest antioxidant and anti-arthritic activities, with an IC_50_ value of 88.39 µg/mL in the DPPH assay and 81.35% inhibition of protein denaturation. Additionally, the compounds derived from the four marine species exhibited significant binding affinity to the selected target receptors, thereby validating the experimental findings. The marine species studied possess multifaceted pharmacological properties, supporting their potential as natural sources for developing therapeutic agents supporting the blue economy. Further studies are recommended to isolate active compounds and elucidate underlying mechanisms to support future drug development efforts.

## 1. Introduction

The increasing demand for medications with fewer side effects has led researchers to explore marine biodiversity as a rich source of bioactive compounds. Such ecosystems harbor organisms capable of producing secondary metabolites with advanced and unique chemical structures and marvelous pharmacological effects [1]. Marine ecosystems are being extensively studied due to their nutraceuticals, functional foods, and pharmaceuticals, which support sustainable health and nutrition solutions.

Approximately nine kilometers off the coast of Cox’s Bazar-Teknaf peninsula lies St. Martin’s Island, the only coral island in Bangladesh. Not only is it known for having the only coral reef in the country, but it also comprises coral reefs, seagrass meadows, and intertidal zones, home to a plethora of marine life [2,3]. The ecological richness of the island makes it an ideal site for marine bioprospecting.

The present study focuses on four marine species found along the west coast of St. Martin’s Island: *Padina australis*, *Holothuria atra*, *Spatoglossum asperum*, and *Hypnea valentiae*. These are gaining scientific attention for bioactive compounds, although they have been historically harvested by coastal communities around the world for their purported health benefits. Gastrointestinal health, skin issues (UV-induced damage and photoaging, acne/propionibacterium acnes–related problems, irritant/pollutant-related skin damage), and infections are only a few of the ailments that it is claimed can be treated with *Padina australis*. *Holothuria atra* is known as the black sea cucumber and is used in folk medicine for various ailments due to its bioactive compounds. It produces triterpene glycosides and mucopolysaccharides that are known to possess antiviral, anti-inflammatory, and cytotoxic activities [4]. *Spatoglossum asperum* shows powerful antioxidant activity. *Hypnea valentiae* is an alga that is valued for its sulfated polysaccharides, which exert strong anticoagulant, antioxidant, and antimicrobial activities [5].

It is scientifically unexplained why there is a lack of research on seaweeds and sea cucumbers, especially when these organisms are deeply rooted in folk medicine. The use of seaweed is documented in the Chinese, Greek, and Japanese medical systems for centuries for the treatment of goiter, chronic inflammatory conditions, and other gastrointestinal diseases, because of its iodine content and healing polysaccharides [4].

Recent studies have shown that compounds originating from the sea are regarded as safer and more eco-friendly options compared to synthetic drugs. Extracts from seaweed and sea cucumbers are now widely used in dietary supplements, skincare products, and functional foods because they are considered a part of the health industry [6]. These natural products are valued as preventive health products due to their potent antioxidant, antimicrobial, anti-aging, and immune-enhancing properties and are especially used in preventive medicine [7,8,9,10,11].

Research shows that phytomedicine taken from marine life helps treat chronic and degenerative ailments. Seaweeds are also rich in bioactive compounds, such as polyphenols, carotenoids, and peptides, that have antioxidant properties. In addition, they also possess anti-inflammatory, anti-obesity, and cancer-fighting capabilities [12,13].

Marine organisms like seaweed and sea cucumbers have immense importance in healthcare practices and the blue economy. Coastal populations commonly use these organisms to fulfill their nutritional and medicinal needs. Even though these organisms are used globally in traditional medicine systems, very little scientific research applies pharmacological principles to these organisms. In light of Bangladesh’s developing blue economy framework, which prioritizes the sustainable use of oceanic resources for employment, economic growth, and biodiversity conservation, closing this gap is crucial [14,15,16]. Although previous studies have demonstrated various biological activities of these marine species comprehensive investigations integrating in vivo, in vitro, and in silico approaches to evaluate their analgesic, anti-inflammatory, antioxidant, and anti-arthritic properties are still lacking [17,18,19].

Our study addresses this research gap by exploring marine organisms as promising sources of novel therapeutic agents. Accordingly, the aims of this study are to screen crude extracts for phytochemicals to identify important bioactive compounds, evaluate the pharmacological characteristics (anti-inflammatory, analgesic potential by in vivo methods and anti-arthritic and antioxidant potential by in vitro methods) of *P. australis*, *H. atra*, *S. asperum*, and *H. valentiae*, which could lead to the creation of safer and more sustainable alternatives to traditional medications. In addition to experimental approaches, we integrated in silico methodologies to strengthen and support our findings. Computational tools such as molecular docking and virtual screening have become essential components of modern drug discovery, enabling the rapid identification and optimization of potential lead compounds while minimizing time, cost, and resource consumption prior to laboratory validation. Therefore, molecular docking, ADME/T profiling, and prediction of activity spectra for substances (PASS) analyses were conducted to further substantiate the therapeutic potential of the identified bioactive compounds and reinforce the pharmacological relevance of the studied marine extracts.

## 2. Materials and Methods

### 2.1. Sample Collection and Preparation of Crude Extract

For the current investigation, *Padina australis* (PA), *Holothuria (Halodeima) atra* (HA), *Spatoglossum asperum* (SPT), and *Hypnea valentiae* (HV) were collected from the west coast of St. Martin’s Island, Bangladesh. The samples were identified by Abu Sayeed Muhammad Sharif, Senior Scientific Officer, Biological Oceanography, Bangladesh Oceanographic Research Institute. The collected samples were separated from undesirable materials and thoroughly washed with seawater, followed by freshwater, to remove surface debris. Then, the samples were sun-dried under a covered shed. The samples were not exposed to direct solar radiation for one week, except *Holothuria (Halodeima) atra.* The samples were ground into a coarse powder using a high-capacity grinding machine (Brand-Damai). About 1240 g of raw tissues from *H. atra* and 480 g, 270 g, and 267 g of powdered samples of *P. australis, S. asperum*, and *H. valentiae*, respectively, were added to a clean, conical flask and soaked in 2000 mL, 1800 mL, 900 mL, and 1000 mL of ethanol, respectively [20]. The containers with their contents were sealed with foil and kept for a period of 15 days, except *H. atra*, which was soaked in ethanol for 72 h, accompanied by occasional shaking and stirring. Each mixture was then filtered through a fresh cotton plug and finally through a Whitman No.1 filter paper. The volume of the filtrate was then reduced using a rotary evaporator (RE200, Bibby Sterilin, Stone, Staffordshire, UK) at 40 °C to obtain the crude ethanol extract [21]. Extracts were stored in airtight vials at 4 °C, protected from light, until further analysis [22]. It is noted that all chemical reagents used in this study were purchased from Merck KGaA (Darmstadt, Germany).

### 2.2. Phytochemical Investigation

The preliminary phytochemical screening of the marine-derived extracts was conducted using standard qualitative assays to identify major classes of secondary metabolites [23].

### 2.3. In Vivo Investigations

#### 2.3.1. Experimental Animals

We obtained 220 Swiss albino mice (30 female and 190 male) from Cumilla University’s animal research department in Bangladesh. They were kept in clean and only dry polypropylene cages with 12 h light–dark cycle at 25 ± 2 °C and 45–55% relative humidity. The mice were fed with a standard laboratory diet and water ad libitum for 14 days. Food was withdrawn 12 h before and during the experiment. As these animals are very sensitive to environmental changes, they were kept before the test for at least 3–4 days in the environment where the experiment would take place. Then, 150 male mice that weighed between 25 and 30 g were used for the main assays (in vivo experiments). Ethical approval was taken from the Institutional Ethics Review Board (IERB), Eastern University, Dhaka, Bangladesh, under Approval No.-IERB-2024-0001.

#### 2.3.2. Acute Toxicity Evaluation and Dose Determination for In Vivo Study

To assess the acute toxicity of each extract, twenty-five overnight-fasted mice, roughly weighing between 25 and 27 g (female non-pregnant mice according to OECD 425 guideline), were divided into five groups of five. Each group received specific oral treatments, such as dosages of each extract ranging from 0.5 to 4 g/kg body weight. Following the oral injection of the extract, the mice were fasted for a further 3–4 h. Mice were examined after oral extract therapy for changes in their eyes, mucous membranes, skin, fur, circulatory rate, respiration rate, and autonomic and central nervous systems. These observations were made for the first 30 min after oral therapy, then every 24 h, with a focus on the first 4 h, and daily thereafter for a total period of 14 days to document any delayed toxicity (such as behavioral changes, and mortality) in accordance with OECD guidelines for acute oral toxicity testing. Body weight of each animal was recorded prior to treatment (Day 0) and subsequently at regular intervals (Day 7 and Day 14) throughout the observation period to assess any treatment-related changes [24,25].

#### 2.3.3. Evaluation of Analgesic Activity

##### Acetic Acid-Induced Writhing Method

The peripheral analgesic potential of the extracts was assessed using the acetic acid-induced writhing model in Swiss albino mice [26]. Fifty mice were randomly allocated into ten groups (n = 5 per group): Group I served as the control (1% Tween-80 and DMSO in saline), Group II received the standard drug diclofenac sodium (50 mg/kg), while Groups III–X received oral doses (200 or 400 mg/kg) of ethanolic extracts of PA, SPT, HA, or HV. All test suspensions were prepared by triturating the respective extract in Tween-80, followed by dilution with saline and vortex mixing. At time zero, all treatments were administered orally via feeding needle. After a 40-min absorption period, 0.7% (*v*/*v*) acetic acid (prepared by diluting 0.7 mL glacial acetic acid to a final volume of 100 mL with distilled water) was injected intraperitoneally. Five minutes post-injection, each mouse was observed for 15 min to count abdominal constrictions (writhes), with partial writhes counted as 0.5. The percentage of inhibition of writhing was calculated using the formula:

$$\%\;ofInhibition=\frac{Nc-Nt}{Nc}\times 100$$

Here,

Nc = number of writhing in control

Nt = number of writhing in test animals 

##### Tail Immersion Test

The central analgesic effect of the extracts was evaluated using the tail immersion method in Swiss albino mice [27,28]. Fifty fasted mice were randomly divided into ten groups (n = 5 per group). Group I received vehicle control (1% Tween-80 and DMSO in saline), Group II was administered the standard drug pentazocine (10 mg/kg), and Groups III–X received oral doses (200 or 400 mg/kg) of PA, SPT, HA, or HV. All test and standard suspensions were prepared in Tween-80 and saline, homogenized using vortex mixing, and adjusted according to individual body weights. A 2–3 cm portion of each mouse’s tail was immersed in a water bath maintained at 50 ± 1 °C. Baseline (predrug) latency was calculated as the mean of three separate measurements per animal, with a 2 min interval between successive trials. Following treatment, reaction latencies were recorded again at 30, 60, 90, and 120 min using the same procedure. Only mice with baseline latencies between 3 and 5 s were included. Reaction time and baseline latency were recorded in seconds (s) using a smartphone stopwatch with a display resolution of 0.01 s. A cut-off time of 15 s was employed to avoid tissue injury. The percentage of maximal possible effect (% MPE) was calculated as:

$$\%\;of\;MPE=\left\{\frac{\left(Postdrug\;latency-Predrug\;latency\right)}{\left(cut\_off\;time\;(15.0\;s)-Predrug\;latency\right)}\right\}\times 100$$

Additionally, the percentage of elongation in latency time relative to the control was determined to assess central analgesic activity:

$$\%\;of\;Elongation=\frac{Latency\;of\;test\;sample-Latency\;of\;control}{Latency\;of\;test\;sample}\times 100\;\;$$

#### 2.3.4. Evaluation of Anti-Inflammatory Activity by Carrageenan-Induced Paw Edema

The anti-inflammatory effect of the extracts was assessed using the carrageenan-induced paw edema model in Swiss albino mice [29,30]. Fifty fasted mice were randomly divided into ten groups (n = 5). Group I served as the control (1% Tween-80 and DMSO in saline), Group II received the standard drug indomethacin (10 mg/kg), while Groups III–X were treated with 200 or 400 mg/kg of PA, SPT, HA, or HV. All test suspensions were prepared by triturating the appropriate amount of extract in Tween-80, followed by the addition of saline and vortexing to ensure uniformity. A total of 45 min after oral administration of the respective treatments, paw edema was induced by subplantar injection of 100 µL of 1% carrageenan solution (prepared by dissolving 0.2 g carrageenan in 20 mL saline) into the right hind paw. Paw circumference was measured using a non-elastic thread before carrageenan injection (0 h) and subsequently at 1, 2, 3, and 4 h post-injection. The change in paw circumference (in cm) was used to evaluate inflammation, and the percentage inhibition of edema was calculated using the formula:

$$(\%)\;Inhibition\;of\;edema\;=\frac{\left(C_{t}-C_{o}\right)control-\left(C_{t}-C_{o}\right)treated}{\left(C_{t}-C_{o}\right)control}\;\times 100$$

Here, C_t_ = Mean paw circumference for each group at different time intervals, C_o_ = Mean paw circumference for each group before carrageenan injection.

### 2.4. In Vitro Investigations

#### 2.4.1. Evaluation of Antioxidant Activity by DPPH Assay

The antioxidant potential of the extracts was evaluated using the 2,2-diphenyl-1-picrylhydrazyl (DPPH) free radical scavenging assay [31,32]. A stock solution of 500 µg/mL was prepared by dissolving 5 mg of each extract in 10 mL of ethanol. Serial dilutions were then performed with methanol to obtain concentrations of 250, 125, 62.5, 31.25, and 15.63 µg/mL. Ascorbic acid (AA) served as the positive control at equivalent concentrations, while DPPH solution without any sample was used as the negative control. Ethanol was used as a blank to calibrate the UV-Vis spectrophotometer. The DPPH solution was freshly prepared by dissolving 4 mg of DPPH in 100 mL of methanol (0.004% *w*/*v*) and stored at 20 °C until use. In the assay, a known volume of sample or control solution was mixed with DPPH and incubated in the dark for 30 min. The absorbance was then measured at 517 nm. The percentage of DPPH scavenging activity was calculated using the formula:

$$\%\;of\;Scavenging\;effect=\;\frac{Absorbance\;of\;Control-Absorbance\;of\;Sample}{Absorbance\;of\;Control\;}\times \;100$$

The median inhibitory concentration (IC_50_), the concentration required to scavenge 50% of the DPPH radicals, was determined by plotting log concentration versus % scavenging activity. Linear regression trendlines were generated, and IC_50_ values were extrapolated using the Quest Graph™ IC_50_ Calculator, an online analysis tool provided by AAT Bioquest, Inc. (Sunnyvale, CA, USA), available at https://www.aatbio.com/tools/ic50-calculator (accessed on 25 September, 2025) based on four-parameter logistic regression models. Graphs and fitted equations were included to ensure reproducibility and transparency.

$$Y=Min+\frac{Max-Min}{1+{\left(\frac{X}{{IC}_{50}}\right)}^{Hill\;coefficient}}$$

#### 2.4.2. Evaluation of Antiarthritic Activity by Bovine Serum Albumin Assay

The in vitro antiarthritic potential of ethanol extracts of PA, HA, SPT, and HV was assessed using the inhibition of protein denaturation method with bovine serum albumin (BSA) [33,34]. Diclofenac sodium served as the reference standard. Test mixtures (0.5 mL) were prepared by combining 0.45 mL of 5% *w*/*v* aqueous BSA with 0.05 mL of extract solution at varying concentrations (62.5, 125, 250, 500, and 1000 µg/mL). Control solutions contained BSA and distilled water, while product control solutions included distilled water and extract. Standard solutions were similarly prepared using diclofenac sodium. All samples were adjusted to pH 6.3 with 1 N HCl and incubated at 37 °C for 20 min, followed by heating at 57 °C for 3 min to induce denaturation. After cooling, 2.5 mL of phosphate buffer was added, and absorbance was measured at 416 nm using a UV-Visible spectrophotometer. The percentage inhibition of protein denaturation was calculated using the formula:

$$\%\;inhibition=\left[\frac{\left(A_{C}-A_{S}\right)}{A_{C}\;}\right]\times 100$$

where Ac represents the absorbance of the control (BSA with distilled water) and As represents the absorbance of the test sample or standard drug.

The control is the value that corresponds to complete protein denaturation, and all results were compared against the standard drug to evaluate the relative antiarthritic efficacy of each extract.

### 2.5. In Silico Investigations

#### 2.5.1. Toxicity Prediction by admetSAR

Since toxicity poses a significant challenge in the development of new medications, the admetSAR online tool (http://lmmd.ecust.edu.cn/admetsar2/, accessed on 25 September 2025, SwissADME, and pkCSM were employed to evaluate the toxicological profiles and pharmacokinetic properties of the identified compounds. These tools assessed parameters such as absorption, distribution, metabolism, excretion, and toxicity (ADMET) to ensure the safety and efficacy of compounds. Additionally, the compounds were screened using the Lipinski Rule of Five, which helps predict bioavailability by evaluating molecular weight, lipophilicity (logP), hydrogen bond donors, and hydrogen bond acceptors. Compounds violating more than one criterion of the Lipinski Rule of Five were excluded to prioritize those with favorable drug-like properties for further investigation [35].

#### 2.5.2. Preparation of Ligands

The structures of 20 different compounds from *Holothuria atra* [36], *Hypnea valentiae* [37], *Padina australis* [38], and *Spatoglossum asperum* [39] were retrieved from the PubChem database (https://pubchem.ncbi.nlm.nih.gov/). Additionally, diclofenac (PubChem CID: 3033) and ascorbic acid (PubChem CID: 135665655) were included as reference standards to compare and contrast the docking performance of the marine-derived compounds. The ligand structures were downloaded in 2D SDF format and subsequently energy-minimized using the PyRx tool to optimize their conformations for docking. Virtual screening was performed using PyRx from MGLTools (https://ccsb.scripps.edu/mgltools/), with all parameters maintained in their default settings to ensure consistency and reliability in identifying the best possible hits for the target proteins [40,41].

#### 2.5.3. Preparation of Proteins

The protein structures of Cyclooxygenase-2 inhibitor (PDB: 6COX), Cyclooxygenase-1 (PDB: 6Y3C), human glutathione reductase (PDB: 1XAN), and TNF-alpha (PDB ID: 2AZ5) were downloaded from the RCSB Protein Data Bank (PDB). These proteins were utilized to evaluate analgesic, anti-inflammatory, antioxidant, and antiarthritic activities, respectively. At the start of the study, ligands bound to the proteins were removed using Discovery Studio Visualizer (BIOVIA edition 2025). The proteins were then optimized and prepared for docking using UCSF Chimera (version 1.18) [42]. During this process, water molecules were removed, polar hydrogen atoms were added to the necessary residues, and Gasteiger charges were estimated. Finally, the prepared protein structures were converted into pdbqt format using AutodockVina (version 1.2.0) for subsequent docking studies [43].

#### 2.5.4. Molecular Docking Study

The protein structures downloaded from the RCSB Protein Data Bank (accessed on 11 September 2024) were utilized for docking computations. The outcomes of the docking studies were analyzed using PyMOL (version 2.6.2), a powerful tool for visualizing and interpreting molecular interactions. These technologies enabled the identification of interaction types—such as hydrogen bonds, π–π stacking, and cation–π interactions—and their contributions to ligand binding. Additionally, PyMOL provided further insights into the detailed interactions between ligands and receptors, facilitating a deeper understanding of the binding mechanisms. This approach aligns with current advances in protein–ligand docking, addressing both the state-of-the-art methodologies and future challenges in the field [44].

### 2.6. PASS Prediction

The prediction of activity spectra for substances (PASS) online tool ‘Way2Drug–PASS Online, available online: https://share.google/suNMcLtvn5Z8KHNxa (accessed on 11 September 2024)’ was utilized to predict the potential biological activities of the selected compounds. The predictions are based on Pa (probability of activity) and Pi (probability of inactivity) values, which range from 0.000 to 1.000. Specific search terms such as “Antinociceptive/Analgesic stimulant,” “Antiinflammatory,” “Antioxidant,” and “JAK2 expression inhibitor” were used to evaluate the analgesic, anti-inflammatory, antioxidant, and antiarthritic potentials of the compounds, respectively. A compound is considered to possess biological potential when its Pa value exceeds its Pi value. Furthermore, the following criteria were applied to assess therapeutic potential: Pa < 0.5 indicates poor pharmaceutical activity, 0.5 < Pa < 0.7 suggests intermediate therapeutic potential, and Pa > 0.7 signifies high medicinal activity [45,46].

### 2.7. Statistical Analysis

All data were expressed as mean ± SEM (standard error of mean). The results were analyzed statistically by one-way ANOVA followed by post hoc Dunnett’s *t*-test using statistical software Statistical Package for Social Science (SPSS, Version 16.0, IBM Corporation, New York, NY, USA). Results * *p* < 0.05, ** *p* < 0.01, and *** *p* < 0.001 were considered statistically significant as compared to control.

## 3. Results

### 3.1. Phytochemical Screening

The detailed results of the chemical investigation carried out with ethanol extracts of PA, SPT, HV, and HA are represented in Table 1. All four extracts showed the presence of key secondary metabolites, including alkaloids, flavonoids, tannins, saponins, glycosides, terpenoids, phenols, and carbohydrates, indicating a broad spectrum of phytoconstituents.

### 3.2. Acute Toxicity Evaluation and Dose Determination for In Vivo Study

Throughout the study, no signs of toxicity such as restlessness, convulsions, impaired motor activity, diarrhea, coma, or lacrimation and significant body changes were observed in any of the test groups at the administered doses. The body changes throughout the observation period is presented in Table 2. The fact that the animals did not die indicates that the LD50 exceeds 4 g/kg of body weight. Based on prior in vivo research involving plant-derived extracts and supported by the outcomes of the acute toxicity assessment, doses of 200 mg/kg and 400 mg/kg were selected as moderate and higher treatment levels, respectively, to explore potential dose-dependent effects.

### 3.3. In Vivo Investigations

#### 3.3.1. Evaluation of Analgesic Activity

##### Acetic Acid Induced Writhing Method

**Figure 1:**
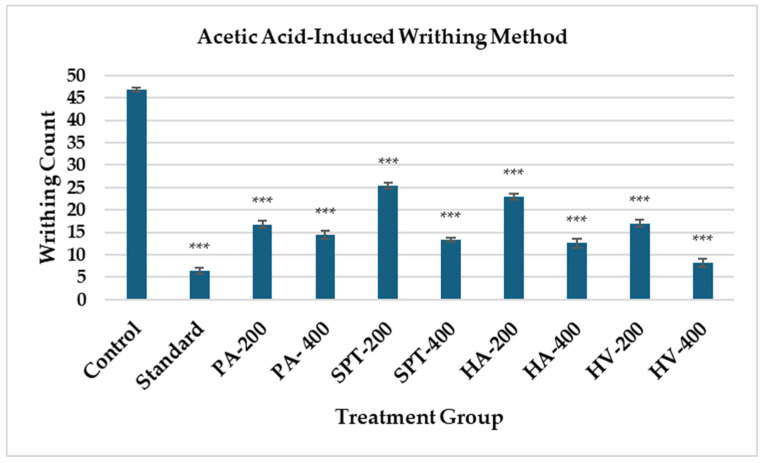
Evaluation of Analgesic Activity of Marine Sample through Acetic Acid-Induced Writhing Method. Data are expressed as mean number of writhes ± SEM (n = 5). Statistical analysis was performed using one-way ANOVA followed by Dunnett’s post hoc test. *p* < 0.001 *** compared with the control group. PA = *Padina australis*, SPT = *Spatoglossum asperum*, HA = *Holothuria atra*, HV = *Hypnea valentiae*.

The peripheral analgesic activity was assessed using the acetic acid-induced writhing method, and the results are presented in Table 3 and Figure 1. The standard group significantly reduced the number of writhes (6.40 ± 0.75), showing 86.35% inhibition (*p* < 0.001). Among the test groups, HV-400 exhibited the highest activity with 8.20 ± 0.86 writhes (82.51% inhibition), followed by HA-400 (12.60 ± 0.93; 73.13%), and SPT-400 (13.40 ± 0.40; 71.43%) (*p* < 0.001). The PA-400 group also showed notable inhibition (14.40 ± 0.93; 69.30%) (*p* < 0.001). All test groups produced a statistically significant reduction in writhing, with higher doses (400 mg/kg) demonstrating superior efficacy compared to their lower-dose counterparts.

##### Tail Immersion Test

The central analgesic effects of marine ethanol extracts were evaluated via the tail immersion method, with pentazocine (10 mg/kg, i.p.) serving as the reference standard. Reaction times were recorded at 30, 60, 90, and 120 min post-treatment (Table 4).

In the control group, tail withdrawal latency remained low throughout the observation period, with a peak response of 1.28 ± 0.15 s at 90 min. In contrast, the standard group exhibited a significant increase in latency, peaking at 60 min (9.84 ± 1.17 s; *p* < 0.001). Among the tested extracts, PA-400 produced the most prominent analgesic effect, with a peak reaction time of 5.16 ± 0.28 s at 90 min (*p* < 0.001). The 200 mg/kg dose of PA also demonstrated significant activity, with peak latency of 3.62 ± 0.27 s at the same timepoint (*p* < 0.001). Remaining samples demonstrated a slight increase in tail-flick latency compared to control, but the results were not statistically significant (*p* > 0.05). The calculated percent elongation of latency time and maximum possible effect (%MPE) supported these observations (Table 5). PA-400 exhibited the highest %MPE at 90 min (28.71%), followed by PA-200 (17.54%). HA-400 and SPT-400 showed moderate MPEs (9.26% and 6.33%, respectively), while HV groups displayed relatively low activity. These results indicate that PA extracts, particularly at higher doses, exhibit significant central analgesic activity, though not as potent as the standard drug.

#### 3.3.2. Evaluation of Anti-Inflammatory Activity by Carrageenan-Induced Paw Edema

The investigated extract showed dose-dependent attenuation in inflammation; the results are depicted in Table 6.

The test compounds demonstrated significant dose- and time-dependent anti-inflammatory effects in the paw edema model. The standard drug indomethacin (10 mg/kg) showed rapid inhibition (86.95% at 1 h, 100% by 3 h; *p* < 0.001). Among the test groups, HV-400 and HA-400 exhibited the most potent early activity, achieving 100% inhibition by the 2nd hour (*p* < 0.001), while PA-400 and SPT-200 and -400 showed progressive effects. Notably, PA-200 initially increased edema (−34.78% at 1 h) but later exerted strong inhibition (94.74% at 4 h; *p* < 0.001). All active treatments maintained sustained anti-inflammatory effects without adverse effects, with higher doses (400 mg/kg) generally producing faster and more pronounced responses than their 200 mg/kg counterparts.

### 3.4. In Vitro Investigations

#### 3.4.1. Evaluation of Antioxidant Activity by DPPH Assay

The antioxidant potential of ethanol extracts of the studied samples was evaluated using the DPPH free radical scavenging assay. The results, including percentage scavenging activity and IC_50_ values, are summarized in Table 7.

**Figure 2:**
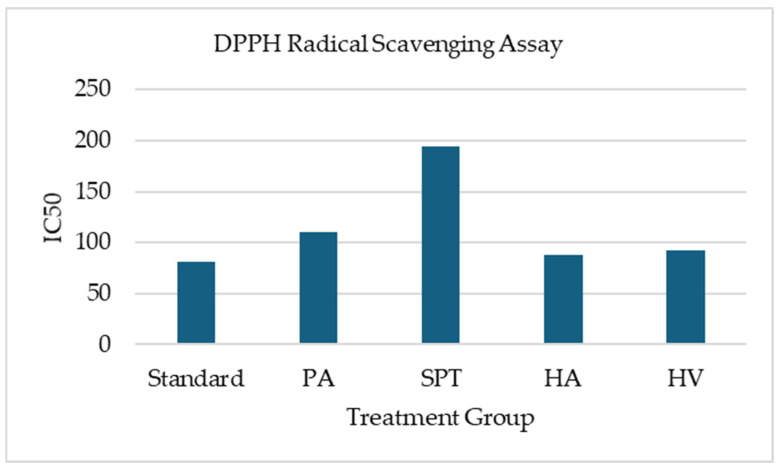
IC_50_ Value of Different Marine Samples in the DPPH Radical Scavenging Assay. PA = *Padina australis*, SPT = *Spatoglossum asperum*, HA = *Holothuria atra*, HV = *Hypnea valentiae*. This assay was conducted as a preliminary screening, and no inferential statistical analysis was applied.

A dose-dependent increase in DPPH scavenging activity was observed for all extracts, with higher concentrations (500 µg/mL) exhibiting the strongest radical scavenging effects. Ascorbic acid, used as the standard antioxidant, demonstrated the highest activity overall, with an IC_50_ value of 80.7 µg/mL. Among the crude extracts, HA showed the most potent antioxidant activity, achieving an IC_50_ of 88.4 µg/mL, closely followed by HV (IC_50_ = 92.6 µg/mL), indicating a strong free radical scavenging capacity. PA also displayed considerable antioxidant efficacy, with an IC_50_ of 110.2 µg/mL. In contrast, SPT showed comparatively lower antioxidant activity, with the highest IC_50_ value (194.6 µg/mL), suggesting a weaker capacity to neutralize DPPH radicals (Figure 2).

The observed variation in IC_50_ values reflects the differences in the phytochemical composition and antioxidant constituents among the tested marine extracts. Notably, all investigated extracts demonstrated significant scavenging activity (>75% inhibition) even at the lowest concentration tested (15.63 µg/mL), highlighting their potential as natural antioxidant sources.

#### 3.4.2. Evaluation of Antiarthritic Activity by Bovine Serum Albumin Assay

The results for the in vitro antiarthritic activity of the extracts are shown in Table 8. All extracts demonstrated concentration-dependent inhibition of protein denaturation in the assay. However, HA and HV exhibited potency comparable to the standard diclofenac sodium. At the highest concentration (1000 µg/mL), HA and HV achieved 81.35% and 80.17% inhibition, respectively, nearing the standard’s efficacy (83.05%). Similarly, at a 500 µg/mL concentration, HA and HV showed similar performance (69.49% vs. 72.88%) as the standard (74.58%), suggesting superior activity at intermediate doses. PA and SPT also showed significant inhibition (80.34% and 76.95% at 1000 µg/mL) but required higher concentrations for effects comparable to HA/HV.

### 3.5. In Silico Investigations

#### 3.5.1. Drug-likeness Analysis

The study evaluated the pharmacokinetic and toxicological properties of 20 compounds derived from four different marine species, *Holothuria atra*, *Hypnea valentiae*, *Padina australis*, and *Spatoglossum asperum*, to assess their potential as drug-like compounds. Each molecule was found to comply with Lipinski’s Rule of Five, indicating favorable oral bioavailability. Lipinski’s criteria ensure that a compound has appropriate molecular weight, hydrogen bond donors, hydrogen bond acceptors, and lipophilicity for effective absorption and oral consumption. To predict the toxicological and pharmacokinetic profiles of these compounds, the admetSAR server (http://lmmd.ecust.edu.cn/admetsar2/, accessed on 11 September 2024), SwissADME (http://www.swissadme.ch/), and the pKCSM online tool (http://biosig.unimelb.edu.au/pkcsm/, accessed on 11 September 2024) were utilized (Table 9). These computational approaches collectively ensured a robust evaluation of the compounds’ suitability for oral administration and their potential toxicological risks, supporting their consideration as promising candidates for further drug development studies.

#### 3.5.2. Docking Analysis

Molecular docking was performed to investigate the interactions between phytochemical elements from the four marine species and their corresponding protein targets. Table 10 presents the combined docking scores for each activity, while Table 11 details the in silico binding affinities and non-bonding interactions of selected phytochemicals. These interactions were analyzed in the context of their analgesic, anti-inflammatory, antioxidant, and antiarthritic activities, offering insights into the potential mechanisms underlying their pharmacological effects. The results highlight the binding efficiencies and molecular interactions that contribute to the observed bioactivities.

##### Docking Analysis for Analgesic Activity

**Figure 3:**
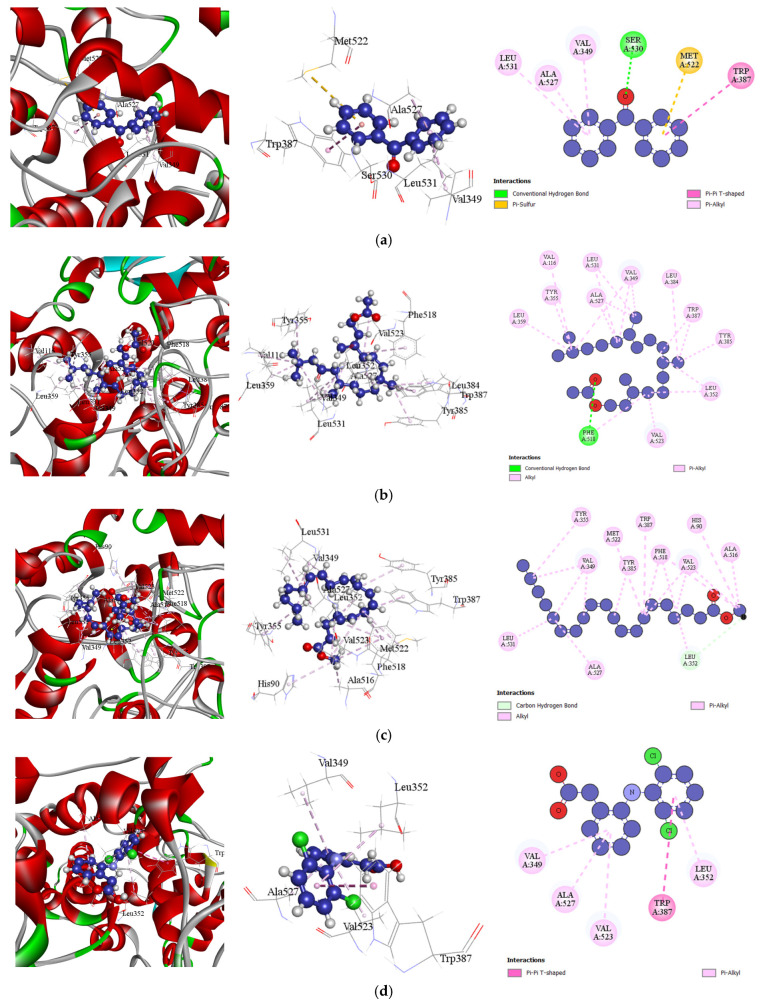
Docking Interactions of Compounds Against Cox-2 Inhibitor (PDB: 6cox): (**a**). Benzophenone (**b**)**.** Phytol acetate (**c**). Methyl arachidonate (**d**). Diclofenac (Standard).

In this study, potential analgesic compounds were screened by docking with the Cyclooxygenase-2 (COX-2) inhibitor (PDB ID: 6COX). The compounds from four different marine extracts exhibited binding affinities ranging from −5.8 to −8 kcal/mol. Notably, Benzophenone from *Spatoglossum asperum* demonstrated the highest binding affinity of −8 kcal/mol. Additionally, Phytol acetate from *Hypnea valentiae* and Methyl arachidonate from *Holothuria atra* showed binding affinities of −7.9 kcal/mol and −7.5 kcal/mol, respectively, which are comparable to the conventional COX-2 inhibitor, Diclofenac (−8.4 kcal/mol). The top-performing compound, Benzophenone, formed six key interactions with amino acid residues SER530, MET522, TRP387, VAL349, ALA527, and LEU531 at short intermolecular distances, indicating strong binding and high affinity for the COX-2 active site [Table 11 (Section 1), and Figure 3]. These findings highlight the potential of these marine-derived compounds as promising analgesic agents.

##### Docking Analysis for Anti-Inflammatory Activity

**Figure 4:**
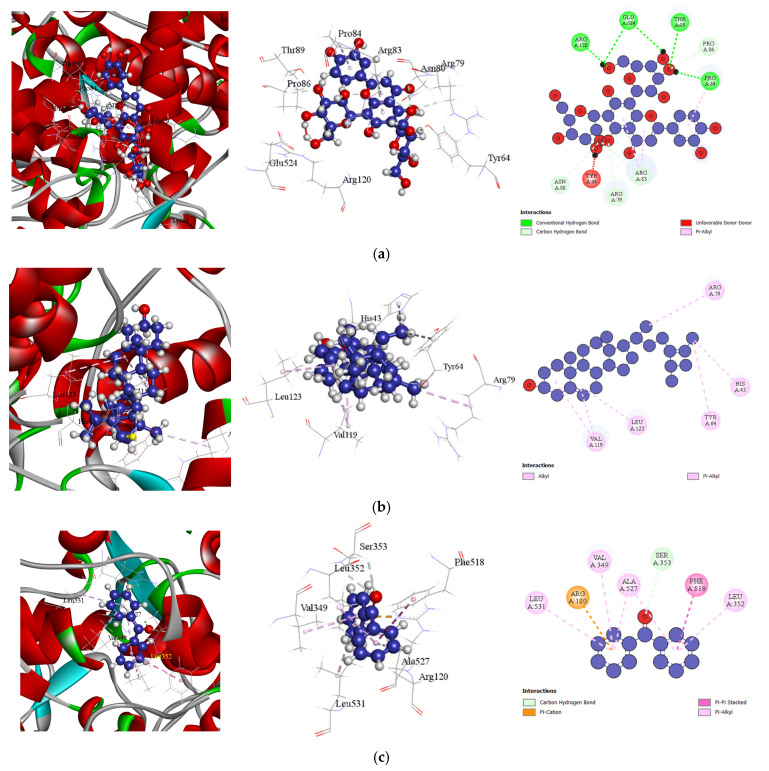

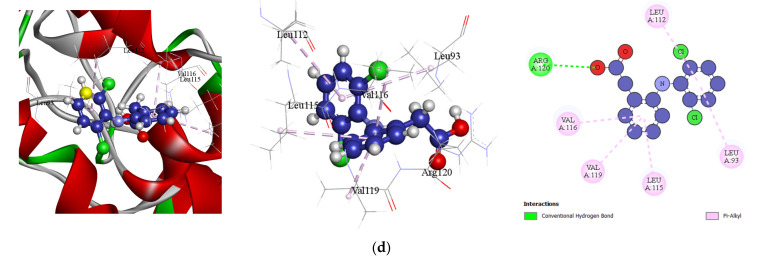
Docking Interactions of Compounds Against Human COX-1 (PDB: 6y3c): (**a**). Lucenin 2 (**b**). Fucosterol (**c**). Benzophenone (**d**). Diclofenac (Standard).

This study also investigated potential anti-inflammatory compounds by screening them against human Cyclooxygenase-1 (COX-1, PDB ID: 6Y3C). The compounds derived from the four different extracts exhibited binding affinities ranging from −4.6 to −8.5 kcal/mol. Notably, Lucenin 2 from *Hypnea valentiae* demonstrated the highest binding affinity of −8.5 kcal/mol. Following this, Fucosterol and Benzophenone showed binding affinities of −8.2 kcal/mol and −7.1 kcal/mol, respectively. The most potent compound, Lucenin 2, formed 11 interactions with key amino acid residues in the active site of the COX-1 receptor, indicating strong binding and high affinity [Table 11 (Section 2), and Figure 4]. These findings suggest that Lucenin 2 and other identified compounds hold significant potential as anti-inflammatory agents.

##### Docking Analysis for Antioxidant Activity

**Figure 5:**
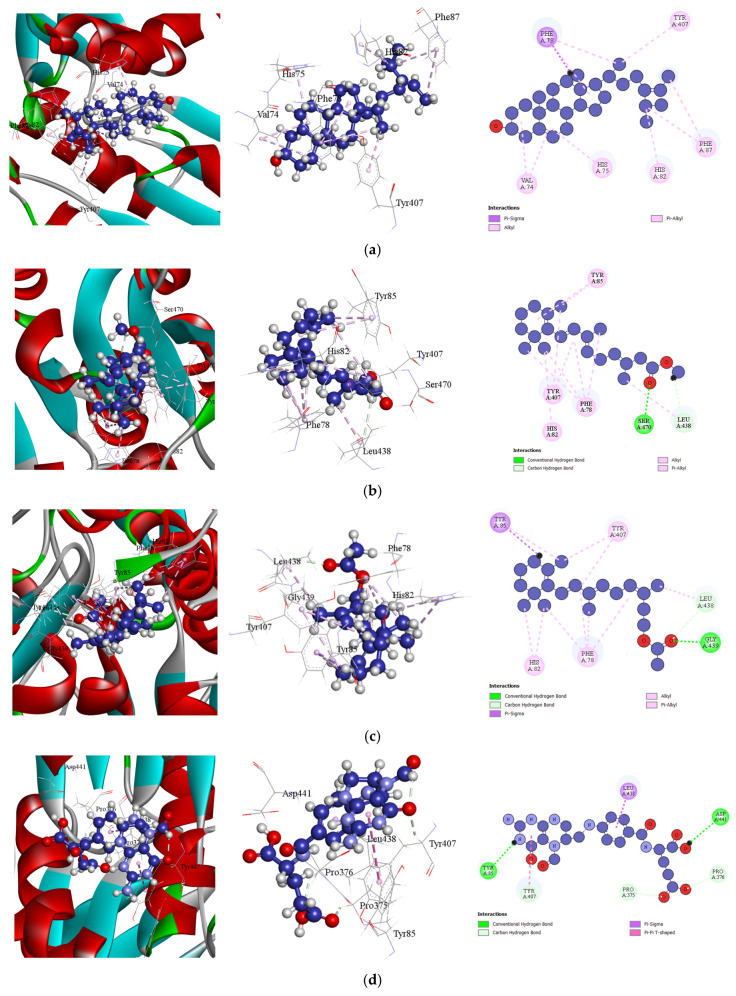
Docking Interactions of Compounds Against Human Glutathione Reductase Inhibitor (PDB: 1xan): (**a**). Fucosterol (**b**). Retinoic acid, methyl ester (**c**). Retinol acetate (**d**). Ascorbic acid (Standard).

The antioxidant potential of selected bioactive compounds from *Holothuria atra*, *Hypnea valentiae*, *Padina australis*, and *Spatoglossum asperum* was evaluated by examining their interactions with the human glutathione reductase inhibitor (PDB: 1xan). All tested compounds demonstrated significant binding affinity to the target receptor. Among them, Fucosterol exhibited the highest binding affinity with a docking score of −7.8 kcal/mol, surpassing retinoic acid, methyl ester, and retinol acetate, both of which showed docking scores of −7.2 kcal/mol. Notably, the standard drug ascorbic acid had a lower binding affinity (−7.3 kcal/mol) compared to Fucosterol. This study revealed that the binding mechanism and interaction pattern of Fucosterol with the receptor were comparable to those of ascorbic acid. A detailed docking analysis indicated that Fucosterol formed ten key interactions with amino acid residues, including PHE78, VAL74 (2), HIS75, PHE78 (2), HIS82, PHE87 (2), and TYR407, at short intermolecular distances [Table 11 (Section 3), and Figure 5]. These interactions highlight the strong affinity of Fucosterol for the active site of the human glutathione reductase inhibitor receptor, underscoring its potential as a promising antioxidant agent.

##### Docking Analysis for Antiarthritic Activity

**Figure 6:**
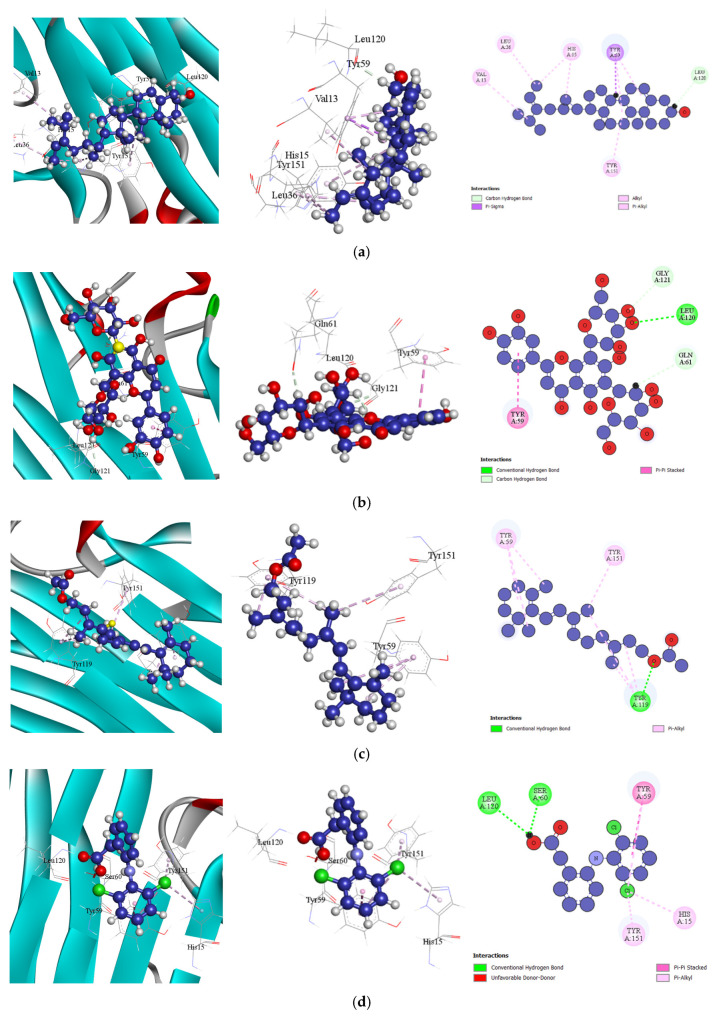
Docking Interactions of Compounds Against TNF-alpha (PDB ID: 2AZ5): (**a**). Fucosterol (**b**). Lucenin 2 (**c**). Retinol acetate (**d**). Diclofenac (Standard).

Each compound exhibiting antiarthritic properties demonstrated significant affinity for TNF-alpha (PDB ID: 2AZ5). Among them, Fucosterol from *Spatoglossum asperum* showed the highest binding affinity of −7.1 kcal/mol, surpassing the standard drug diclofenac (−5.8 kcal/mol). The next two compounds, Lucenin 2 and retinol acetate, both exhibited binding affinities of −6.1 kcal/mol, which were also higher than that of diclofenac. A detailed analysis of the docking process revealed that Fucosterol formed nine key interactions with amino acid residues in the target receptor’s active site, including LEU120, TYR59, LEU36, VAL13, HIS15 (2), TYR59 (2), and TYR151, at short intermolecular distances [Table 11 (Section 4), and Figure 6]. These interactions indicate a strong binding affinity of Fucosterol for TNF-alpha, highlighting its potential as a promising antiarthritic agent.

#### 3.5.3. PASS Prediction

A total of 20 carefully selected compounds from four different marine species were evaluated for their analgesic, anti-inflammatory, antioxidant, and antiarthritic properties using the prediction of activity spectra for substances (PASS) online tool. The findings demonstrated that compounds with significant molecular potency exhibited Pa values greater than their corresponding Pi values, indicating a higher likelihood of biological activity for these compounds (Table 12).

## 4. Discussion

The phytochemical screening of ethanol extracts from PA, SPT, HA, and HV revealed a diverse array of secondary metabolites, suggesting their pharmacological relevance. The consistent presence of alkaloids, flavonoids, phenols, and saponins across all samples underscores the therapeutic potential of these marine-derived species. Alkaloids were detected in all extracts, aligning with reports that marine algae and echinoderms often produce alkaloid compounds with significant analgesic, cytotoxic, and antimicrobial properties [47]. Similarly, flavonoids, abundant in all four extracts, are well-known antioxidants and have demonstrated anti-inflammatory and anticancer activities in marine sources [48]. Their presence across multiple assays in this study confirms their chemical abundance and stability in ethanolic media. Phenolic compounds, also present in all samples, are key contributors to antioxidant defense mechanisms. Previous studies have established the phenolic content of brown algae such as *Padina* and *Sargassum* species to be strongly correlated with radical scavenging activity [49]. Terpenoids, likewise detected in all extracts, are widely recognized for their anti-inflammatory, anticancer, and antiviral effects, and are frequently reported in brown and red algae [50]. The consistent presence of glycosides and saponins further supports the potential of these extracts in modulating membrane integrity and metabolic signaling, consistent with earlier reports of bioactive saponins in marine algae and echinoderms [51,52]. Interestingly, cardiac glycosides were found exclusively in *Padina australis*, which is consistent with findings that certain brown algae may biosynthesize digitalis-like compounds [53]. Quinones and phlobatannins were exclusive to SPT, indicating distinct metabolic capabilities that warrant further investigation. The absence of some constituents in specific extracts (e.g., tannins and reducing sugars in HA) could be attributed to species-specific metabolic pathways or solvent selectivity during extraction. These results emphasize the importance of both species identity and extraction methodology in determining phytochemical yield and profile.

The acetic acid-induced writhing test is a well-established model for evaluating peripheral analgesic activity in rodents. The writhing response is induced by the release of endogenous mediators such as prostaglandins (particularly PGE_2_ and PGF_2_α), bradykinin, histamine, and substance P, which sensitize nociceptors and trigger pain perception [54]. In this study, all tested extracts significantly reduced the number of writhes compared to the control group, indicating promising analgesic potential. The significant inhibition of this nociceptive behavior suggests that the ethanol extracts may interfere with the biosynthesis or action of these inflammatory mediators. Such an effect is indicative of peripheral analgesic activity, possibly through modulation of the cyclooxygenase and lipoxygenase pathways [28]. The higher efficacy observed at 400 mg/kg doses across all extract types further supports a dose-dependent relationship. Overall, the results validate the potential of the studied marine-derived ethanol extracts as peripheral analgesic agents. Comparable literature-based findings reinforce our experimental results. *Holothuria atra* extracts have not been widely evaluated in writhing assays, but *Holothuria grisea* lectin (HGA) exhibits substantial inhibition (~60–70%) of acetic acid-induced writhing at doses between 0.1 and 10 mg/kg, demonstrating potent peripheral analgesic potential [55,56,57]. Data for *Spatoglossum* and *Hypnea* genus members remain limited, but outcomes from brown algae writhing studies suggest analogous analgesic effects [58].

The tail immersion test, which assesses centrally mediated analgesic activity via thermal nociception, is selective for compounds that exert analgesic effects through central mechanisms, particularly via opioid receptor activation. The increased tail withdrawal latency observed with PA suggests modulation of central nociceptive pathways, potentially involving μ-opioid receptors or related neurotransmitter systems such as GABAergic or serotonergic pathways [28]. Previous studies on tail immersion on *Padina australis*, *Spatoglossum asperum*, and *Hypnea valentiae* extract are limited. However, in one study, methanol extracts of *Holothuria atra* showed ~84% inhibition in the tail immersion test at 100–200 mg/kg (p.o.), indicating strong central analgesic potential [59]. In our study, though the reaction time increased gradually for the *Holothuria atra* extract, the result was not statistically significant. This may be due to differences in the extraction method, phytochemical composition, or plant collection season; variations in dose selection or route of administration; biological variability or species/strain sensitivity; and differences in experimental conditions, such as water temperature calibration or timing precision during latency measurement. It is also possible that the sample contains active constituents in subthreshold concentrations for central analgesia in the current dose range. Further optimization of dosage and bioavailability enhancement strategies may be required to validate the central analgesic potential observed in previous reports. Despite the lack of statistical significance, the observed trend aligns with literature findings, warranting further investigation using larger sample sizes or complementary nociceptive models to determine central analgesic efficacy conclusively.

Phytochemical screening of the marine samples confirmed the presence of flavonoids, alkaloids, saponins, and tannins, classes of compounds known to contribute to antinociceptive effects, which further substantiate the findings of analgesic activity [60,61,62]. These constituents likely work synergistically to produce the moderate but significant analgesic effects observed in this test.

Anti-inflammatory activity by carrageenan-induced paw edema experimental model is widely used to evaluate acute inflammation and involves a biphasic response: the early phase (from 0–2 h) is predominantly mediated by the release of histamine, serotonin, and bradykinin, while the late phase (from 2–3 h) is associated with prostaglandins and leukotrienes derived from arachidonic acid metabolism [63]. In the current study, all the extracts at both doses except PA-200 achieved inhibition starting from 1 h, indicating strong suppression of the inflammatory mediators responsible for both phases. This may suggest that these extracts interfere with the release of histamine, serotonin, and bradykinin, and cyclooxygenase activity or modulate pro-inflammatory cytokines such as TNF-α, IL-1β, and IL-6, which are pivotal in amplifying the inflammatory cascade [64]. The observed inhibition of paw swelling suggests potential membrane-stabilizing properties or suppression of vascular permeability, as edema formation in this model results from extravasation of plasma components in response to inflammatory mediators [63]. Given that inflammation is a complex interplay between cellular signals, vascular changes, and cytokine activity, the efficacy of the extracts points to their possible role in modulating multiple targets within this network. The presence of one or combination of phytochemicals such as alkaloid, tannin, steroids or phytosterols, terpenoids, flavonoids, saponins, curcumin, gingerols, resveratrol, quercetin, lycopene, capsaicinoids or capsaicin, salicylates, lignans, polyphenols such as epigallocatechin gallate (EGCG), anthocyanins or proanthocyanidins, omega-3 fatty acids, boswellic acid, sulforaphane, berberine, astaxanthin, ellagic acid, piperine, allicin, betulinic acid, and silibinin have been reported to possess anti- inflammatory activities [65,66,67]. Thus, the presence of some of these chemicals in the investigated extract might be responsible for the anti-inflammatory effect.

The anti-inflammatory activity observed in our study aligns well with previously reported findings on similar marine species. Methanolic extracts of *Holothuria atra* have been shown to significantly reduce carrageenan-induced paw edema in rats in a dose-dependent manner, particularly at 100 and 200 mg/kg doses, which supports the potent anti-inflammatory response observed for *Holothuria atra* in our study [68]. Similarly, *Padina tetrastromatica*, a closely related species to *Padina australis*, demonstrated strong anti-edema effects through its sulfated polysaccharide, ascophyllan, which also modulated pro-inflammatory cytokines such as TNF-α and IL-6 [68]. These results are comparable to the inhibition levels observed with our *Padina australis* extract. Furthermore, previous studies combining *Holothuria scabra* and *Spirulina platensis* also revealed significant suppression of paw swelling in the carrageenan model, supporting the observed efficacy of our SPT extract. In addition, sulfated polysaccharides isolated from *Padina boergesenii* and other brown algae have shown comparable anti-inflammatory effects, further corroborating the potential of SPT and HV in reducing acute inflammation [69,70]. Taken together, these literature reports substantiate the anti-inflammatory efficacy of our tested marine samples and validate the consistency of our findings with prior pharmacological studies.

The DPPH assay, a rapid and reliable method for evaluating free radical scavenging capacity, revealed concentration-dependent antioxidant activity across all marine extracts. The antioxidant activity observed in our study is well supported by previous reports on the same marine species. Methanolic extract of *Holothuria atra* demonstrated strong DPPH radical scavenging activity with up to 84.66% inhibition, indicating potent antioxidant potential [59]. Similarly, *Spatoglossum asperum* and *Hypnea valentiae* extracts showed significant antioxidant activity across multiple assays, including DPPH, ABTS, and superoxide radical scavenging, with IC_50_ values ranging from ~33 to 67 µg/mL [71,72]. In addition, *Padina australis* extract exhibited significant DPPH radical scavenging, measured by an IC_50_ of 267.1 ppm [73]. These findings are in line with the strong antioxidant responses recorded in our current investigation.

These antioxidative effects may result from the presence of phenolic compounds, flavonoids, tannins, alkaloids, saponins, carotenoids, and vitamins, which act through diverse mechanisms including hydrogen atom transfer (HAT), single electron transfer (SET), and metal ion chelation. Flavonoids and polyphenols stabilize free radicals by donating hydrogen atoms or electrons, forming resonance-stabilized antioxidant radicals [74]. Tannins and saponins may inhibit lipid peroxidation by chelating iron or other transition metals involved in reactive oxygen species (ROS) generation [75]. Moreover, carotenoids such as β-carotene and astaxanthin quench singlet oxygen and peroxyl radicals in cellular membranes, protecting against oxidative stress [76]. Given their bioactive richness and natural origin, these marine extracts may serve as promising ingredients in functional foods or nutraceutical formulations aimed at managing oxidative stress and promoting health. Their incorporation into dietary supplements, beverages, or antioxidant-enriched foods could offer preventive benefits against chronic disorders linked to oxidative damage, such as cardiovascular disease, neurodegeneration, and aging-related complications.

The antioxidant activity observed in the present study differs from that reported for the ethyl acetate fraction of *Padina boergesenii*, where a maximum DPPH scavenging activity of 54 ± 1% at 3 mg/mL and an IC_50_ value of 2.6 ± 1.2 mg/mL were reported [77]. In contrast, the ethanol extract of *Padina australis* in the present study exhibited a lower IC_50_ value (IC_50_ 110.20 μg/mL or 0.1101 mg/mL), indicating comparatively stronger antioxidant potential. This variation may be attributed to differences in species, extraction solvent, and phytochemical composition, as ethyl acetate selectively extracts medium-polarity compounds, whereas ethanol extracts a broader range of phenolics and flavonoids [78]. Several studies have demonstrated that the antioxidant activities of seaweed extracts correlate strongly with their total phenolic and flavonoid contents, indicating that these compounds contribute significantly to free-radical scavenging and overall antioxidant capacity [79,80]. So, it can be concluded that the higher antioxidant activity of *Padina australis* in comparison to *Padina boergesenii* is related to the solvent used, which ultimately extracted a wide range of flavonoids and phenolic compounds responsible for the antioxidant effect.

Variability in antioxidant activity among brown seaweeds has been widely reported and is strongly influenced by biochemical composition, geographic origin, and reproductive status. Extracts of *Fucus distichus* collected from different Arctic and sub-Arctic regions have shown marked differences in DPPH radical scavenging capacity. For example, samples from the Barents Sea exhibited the lowest polyphenol content (24.4 mg g^−1^ DW) and the weakest antiradical power (ARP = 1.2 mL mg^−1^), whereas specimens from the White Sea displayed substantially higher polyphenol (and flavonoid) levels and correspondingly stronger antioxidant activity (ARP = 2.2 mL mg^−1^). A similar trend was observed in flavonoid-based assays, with the White Sea samples showing the highest flavonoid concentration (18.4 mg g^−1^ DW) and superior scavenging capacity. Interestingly, a weak negative correlation between fucose- or xylose-containing polysaccharides and antiradical activity was reported, suggesting that phenolic compounds, rather than structural carbohydrates, are the dominant contributors to antioxidant potential in *Fucus* species [81]. Similarly, *Fucus spiralis* from different locations showed significant antioxidant activity that correlated with phenolic content [82]. A recent study revealed higher phenolic content of the ethyl acetate fraction of *Padina australis* (807.20 mg GAE.g^−1^) [77]. In our study, the ethanol extract of *Padina australis* also confirmed the presence of phenolic content in all assays performed, thus justifying the higher antioxidant efficacy. Comparable patterns have been documented in *Ascophyllum nodosum*, where antioxidant activity varies significantly with both geographic origin and reproductive phase. Recent studies demonstrated that *A. nodosum* extracts collected from the Irminger Sea and Norwegian Sea during the sterile phase exhibited the lowest IC_50_ values in DPPH assays, whereas samples from the Barents Sea collected during the fertile phase showed markedly reduced scavenging efficiency. The enhanced antioxidant activity during the sterile phase was closely associated with increased total polyphenol and flavonoid contents, reinforcing the central role of phenolic metabolites in free-radical neutralization [83,84]. Taken together, these observations strongly suggest that ecological conditions, seasonal or reproductive status, and associated biochemical adjustments critically shape antioxidant capacity in brown seaweeds.

*Padina species* are known to exhibit seasonal and environmentally driven variation in growth and biochemical properties. Seasonal surveys of macroalgal assemblages, including *Padina spp.* in tropical coastlines, have documented significant seasonal changes in biomass and physiological status, suggesting an active growth phase post-monsoon, when nutrient levels are elevated, and environmental conditions are favorable for macroalgal metabolism and reproduction [85]. Although reproductive structures of *Padina australis* were not microscopically confirmed in the present study, the October (post-monsoon) collection from the Bay of Bengal coincides with periods of enhanced growth and physiological activity in brown algae, which has been associated with higher metabolic rates and compound accumulation in related species. This environmental influence on physiology likely contributed to increased phenolic compound levels and the observed antioxidant activity, consistent with patterns reported in other brown seaweeds where reproductive or active phases align with biochemical peaks. The strong association between phenolic abundance and radical scavenging activity observed in *Fucus* spp. and *Ascophyllum nodosum* aligns well with our phytochemical screening results and further substantiates the conclusion that phenolic compounds are key determinants of antioxidant potential in marine macroalgae.

Protein denaturation, associated with chronic inflammatory conditions like rheumatoid arthritis, leads to autoantigen production and subsequent immune activation. Inhibiting this process is a widely accepted mechanism for assessing antiarthritic potential in vitro. Phytochemicals such as flavonoids, tannins, alkaloids, saponins, terpenoids, and polyphenols, previously identified in these marine samples, are known to suppress inflammatory mediators by stabilizing proteins, scavenging free radicals, and modulating cytokine expression [86,87]. Furthermore, marine-derived compounds have gained attention due to their diverse pharmacological profiles and minimal side effects. The strong inhibition observed at lower doses, particularly with *Holothuria atra* and *Hypnea valentiae*, supports their therapeutic potential. These findings warrant further in vivo studies to identify bioactive constituents and validate clinical relevance. Marine species-based formulations could thus serve as promising antiarthritic agents, offering safer alternatives to conventional nonsteroidal anti-inflammatory drugs. To the best of our knowledge, no previous studies have reported protein denaturation assay results (anti-arthritic activity) specifically for *Holothuria atra*, *Spatoglossum asperum*, *Hypnea valentiae*, or *Padina australis*. Our findings, therefore, provide novel insight into the anti-arthritic potential of these marine species using this model. Although direct literature on these exact species is absent, broader investigations into brown algae-derived sulfated polysaccharides and sea cucumber lectins have demonstrated significant inhibition of protein denaturation in similar in vitro assays, supporting the plausibility of our results [33,88]. Consequently, our data align with general marine-derived bioactive profiles, highlighting a previously undocumented activity in the four studied species.

Molecular docking, a bioinformatics-based approach, predicts how molecules interact to form stable adducts, providing insights into binding energy, free energy, and stability. This technique is increasingly vital in rational drug design, supporting biological testing by identifying potential therapeutic agents. In this study, key compounds from four marine species (*Holothuria atra*, *Hypnea valentiae*, *Padina australis*, and *Spatoglossum asperum*) were docked against protein targets COX-2 (PDB: 6COX), COX-1 (PDB: 6Y3C), human glutathione reductase (PDB: 1XAN), and TNF-alpha (PDB ID: 2AZ5) to evaluate their analgesic, anti-inflammatory, antioxidant, and antiarthritic activities, respectively. The docking scores ranged from −3.8 to −8.5 kcal/mol, indicating strong interactions between the compounds and their respective protein targets. Notably, these results suggest that the bioactive compounds from the marine species exhibit significant potential for drug design and discovery. The PASS prediction data further corroborated these findings, reinforcing the therapeutic relevance of the identified compounds for treating pain, inflammation, oxidative stress, and arthritis.

## 5. Conclusions

This study provides compelling evidence of the diverse phytochemical and pharmacological potential of marine-derived ethanol extracts from *Padina australis*, *Spatoglossum asperum*, *Holothuria (Halodeima) atra*, and *Hypnea valentiae*. The tested extracts demonstrated promising activities across multiple domains, including antioxidant, anti-inflammatory, analgesic, and anti-arthritic effects, highlighting their therapeutic relevance and broad-spectrum bioefficacy. Molecular docking studies further supported these findings by suggesting potential interactions of the bioactive compounds with key target proteins, reinforcing the observed biological activities. These findings not only validate the traditional medicinal relevance of marine species but also underscore their potential as natural sources for pharmacotherapeutic agents. Overall, this research supports global initiatives aimed at marine conservation and blue economic growth. By blending traditional knowledge with modern pharmacology, the study lays the groundwork for developing marine-derived products that are both effective and environmentally friendly. However, this study has several limitations: the active compounds were not fully isolated, detailed mechanistic pathways were not explored, advanced in vivo models were not used, formulation development was not carried out, and comprehensive toxicological and clinical evaluations are still needed. Future studies addressing these aspects will be essential to confirm the therapeutic potential of these extracts.

## Figures and Tables

**Table 1:** Result of phytochemical screening of PA, SPT, HA, and HV.

Serial No.	Tests for Bioconstituents	Test Names	PA	SPT	HA	HV
1	Alkaloid	(a) Wagner’s Test	+	+	+	+
		(b) Mayer’s Test	+	+	+	+
		(c) Dragendroff’s test	+	+	+	+
		(d) Hager’s test	−	+	+	+
2	Tannin	(a) Braymen’s Test	+	+	−	+
		(b) 10% sodium hydroxide test	+	+	−	+
3	Phytosterol	(a) Salkowski’s test	+	+	+	+
4	Terpenoids	(a) Chloroform and sulfuric acid test	+	+	+	+
5	Flavonoids	(a) Zinc-hydrochloric acid reduction test	+	+	+	+
		(b) Lead acetate test	+	+	+	+
		(c) Alkaline reagent test	+	+	+	+
		(d) Conc. sulfuric acid test	+	+	+	+
		(e) 10% Ferric Chloride test	−	+	+	+
6	Saponins	(a) Sodium bicarbonate	+	+	+	+
		(b) Olive oil test	+	+	+	+
7	Glycosides	(a) Aqueous Sodium hydroxide test	+	+	+	+
8	Cardiac glycosides	(a) Keller–Killiani test	+	−	−	−
9	Quinones	(a) Conc. Hydrochloric acid test	−	+	−	−
10	Phenol	(a) 5% Ferric chloride test	+	+	+	+
		(b) Lead acetate test	+	+	+	+
		(c) Potassium dichromate test	+	+	+	+
11	Reducing sugar	(a) Fehling’s test	+	+	−	+
12	Carbohydrate	(a) Test for starch	+	+	+	+
13	Protein and amino acid	(a) Xanthoproteic test	+	+	+	+
14	Carboxylic acid	(a) Effervescence test	+	+	+	+
15	Phlobatannin	(a) Hydrochloride test	−	+	−	−

Note: Presence (+), Absence (−). PA = *Padina australis*, SPT = *Spatoglossum asperum*, HA = *Holothuria atra*, HV = *Hypnea valentiae*.

**Table 2:** Effect of selected marine samples on mice weight for acute toxicity study.

Group	Dose (g/kg)	Day 0 (g)	Day 7 (g)	Day 14 (g)
Control (1% CMC gel)	—	26.0 ± 0.6	26.5 ± 0.7	26.9 ± 0.6
PA	4.0	25.9 ± 0.2	26.4 ± 0.2	26.9 ± 0.2
SPT	4.0	26.2 ± 0.3	26.7 ± 0.3	27.1 ± 0.2
HA	4.0	25.8 ± 0.2	26.3 ± 0.2	26.6 ± 0.1
HV	4.0	26.2 ± 0.8	26.8 ± 0.7	27.1 ± 0.6

Note: PA = *Padina australis*, SPT = *Spatoglossum asperum*, HA = *Holothuria atra*, HV = *Hypnea valentiae,* CMC = Carboxymethyl cellulose. All values are represented as Mean ± SEM, where n = 5.

**Table 3:** Screening of peripheral analgesic activity by acetic acid-induced writhing method.

Group	Number of Writhes (Mean ± SEM)	% Inhibition of Writhing
Control (1% Tween-80 and DMSO in saline)	46.9 ± 0.5	0
Standard (Diclofenac)	6.4 ± 0.8 ***	86.35
PA-200	16.8 ± 0.8 ***	64.18
PA-400	14.4 ± 0.9 ***	69.30
SPT-200	25.4 ± 0.8 ***	45.84
SPT-400	13.4 ± 0.4 ***	71.43
HA-200	23.0 ± 0.7 ***	50.96
HA-400	12.6 ± 0.9 ***	73.13
HV-200	17.0 ± 0.7 ***	63.75
HV-400	8.2 ± 0.9 ***	82.51

Note: All values are mean ± SEM and statistically analyzed using one-way analysis of variance (ANOVA) followed by Dunnett’s multiple comparison test, n  =  5. *** *p* < 0.001 were considered statistically significant as compared to control. PA = *Padina australis*, SPT = *Spatoglossum asperum*, HA = *Holothuria atra*, HV = *Hypnea valentiae*.

**Table 4:** Reaction times (mean ± SEM) of mice in tail immersion test after administration of test samples.

Sample	Pretreatment	30 min	60 min	90 min	120 min
Control (1% Tween-80 and DMSO in saline)	0.55 ± 0.05	0.99 ± 0.14	1.16 ± 0.11	1.28 ± 0.15	0.99 ± 0.13
Standard (Pentazocin)	3.10 ± 0.15	9.05 ±1.12 ***	9.84 ± 1.17 ***	9.29 ± 1.04 ***	9.13 ±1.17 ***
PA-400	1.19 ± 0.05	1.47 ± 0.07	1.28 ± 0.09	5.16 ± 0.28 ***	1.69 ± 0.16
PA-200	1.20 ± 0.07	1.61 ± 0.10	3.46 ± 0.39 **	3.62 ± 0.27 ***	1.47 ± 0.12
HV-400	1.20 ± 0.07	1.63 ± 0.09	1.26 ± 0.13	1.57 ± 0.20	1.41 ± 0.26
HV-200	1.10 ± 0.04	1.34 ± 0.03	1.54 ± 0.12	1.41 ± 0.15	1.64 ± 0.18
SPT-400	0.57 ± 0.06	1.18 ± 0.04	1.40 ± 0.04	1.49 ± 0.09	1.56 ± 0.15
SPT-200	0.85 ± 0.06	1.28 ± 0.06	1.47 ± 0.03	1.34 ± 0.08	1.36± 0.24
HA-400	0.62 ± 0.04	1.27 ± 0.05	1.40 ± 0.09	1.95 ± 0.11	2.01 ± 0.23
HA-200	0.92 ± 0.17	1.07 ± 0.04	1.36 ± 0.04	1.40 ± 0.07	2.20 ± 0.33

Note: All values are mean ±  SEM and statistically analyzed using one-way analysis of variance (ANOVA) followed by Dunnett’s multiple comparison test, n  =  5. ** *p* < 0.01 and *** *p* < 0.001 were considered statistically significant as compared to control. PA = *Padina australis*, SPT = *Spatoglossum asperum*, HA = *Holothuria atra*, HV = *Hypnea valentiae*.

**Table 5:** Percent elongation of latency time and maximum possible effect after administration of all test samples.

Sample	% of Elongation of Latency Time				% of MPE			
	30 min	60 min	90 min	120 min	30 min	60 min	90 min	120 min
1. Control (1% Tween-80 and DMSO in saline)	-	-	-	-	3.10	4.21	5.06	3.09
2. Standard (Pentazocin)	88.97	88.23	86.20	89.09	50.03	56.64	52.03	50.70
3. PA-400	32.11	9.25	75.13	41.20	2.01	0.61	28.71	3.63
4. PA-200	38.09	66.53	64.58	32.06	2.99	16.38	17.54	1.93
5. HV-400	38.77	8.24	18.55	29.36	3.11	0.45	2.71	1.52
6. HV-200	25.74	24.81	9.21	39.27	1.73	3.14	2.22	3.86
7. SPT-400	15.42	17.40	13.73	36.15	4.21	5.75	6.33	6.85
8. SPT-200	22.03	21.22	4.47	26.76	3.04	4.38	3.48	3.60
9. HA-400	21.41	17.28	34.39	50.35	4.51	5.41	9.26	9.62
10. HA-200	6.73	14.60	8.43	54.73	1.06	3.10	3.41	9.09

PA = *Padina australis*, SPT = *Spatoglossum asperum*, HA = *Holothuria atra*, HV = *Hypnea valentiae*.

**Table 6:** Screening of anti-inflammatory activity by measuring individual paw circumference.

Group	Dose (mg/kg)		Mean Paw Circumference (in cm) ± SEM (%of Inhibition)			
		0 h	1st h	2nd h	3rd h	4th h
Control (1% Tween-80 and DMSO in saline)	-	1.36 ± 0.10	1.82 ± 0.12	1.82 ± 0.12	1.78 ± 0.11	1.74 ± 0.10
Standard (Indomethacin)	10	1.2 ± 0.03	1.26 ± 0.05 *** (86.95)	1.24 ± 0.05 *** (91.30)	1.2 ± 0.03 *** (100)	1.2 ± 0.03 *** (100)
PA-200	200	1.2 ± 0.03	1.82 ± 0.04 (−34.78)	1.44 ± 0.05 ** (47.83)	1.28 ± 0.04 *** (80.95)	1.22 ± 0.04 *** (94.74)
PA-400	400	1.2 ± 0.05	1.52 ± 0.04 * (30.43)	1.38 ± 0.04 *** (60.87)	1.26 ± 0.05 *** (85.71)	1.2 ± 0.05 *** (100)
HV-200	200	1.44 ± 0.04	1.62 ± 0.05 (60.87)	1.48 ± 0.05 * (91.30)	1.44 ± 0.02 ** (100)	1.44 ± 0.04 ** (100)
HV-400	400	1.2 ± 0.05	1.32 ± 0.06 *** (73.91)	1.2 ± 0.05 *** (100)	1.2 ± 0.05 *** (100)	1.2 ± 0.05 *** (100)
SPT-200	200	1.28 ± 0.04	1.54 ± 0.04 * (43.48)	1.4 ± 0.03 *** (73.91)	1.28 ± 0.04 *** (100)	1.28 ± 0.04 *** (100)
SPT-400	400	1.26 ± 0.04	1.52 ± 0.06 * (43.48)	1.4 ± 0.05 *** (69.56)	1.32 ± 0.05 *** (85.71)	1.26 ± 0.04 *** (100)
HA-200	200	1.16 ± 0.05	1.32 ± 0.07 *** (65.22)	1.2 ± 0.07 *** (91.30)	1.16 ± 0.05 *** (100)	1.16 ± 0.05 *** (100)
HA-400	400	1.42 ± 0.06	1.54 ± 0.07 * (73.91)	1.42 ± 0.06 *** (100)	1.42 ± 0.06 *** (100)	1.42 ± 0.06 ** (100)

Note: All values are mean ±  SEM and statistically analyzed using one-way analysis of variance (ANOVA) followed by Dunnett’s multiple comparison test, n  =  5. * *p* < 0.05, ** *p* < 0.01 and *** *p* < 0.001 were considered statistically significant as compared to control. PA = *Padina australis*, SPT = *Spatoglossum asperum*, HA = *Holothuria atra*, HV = *Hypnea valentiae*.

**Table 7:** DPPH Scavenging Activity and IC_50_ Values of Standard and Marine Extracts.

Sample	IC_50_ (µg/mL)	Maximum Scavenging Effect (%)
Standard (Ascorbic acid)	80.7	86.22
PA	110.2	94.35
SPT	194.6	97.84
HA	88.4	92.28
HV	92.6	94.81

PA = *Padina australis*, SPT = *Spatoglossum asperum*, HA = *Holothuria atra*, HV = *Hypnea valentiae*. This assay was conducted as a preliminary screening, and no inferential statistical analysis was applied.

**Table 8:** Antiarthritic activity of ethanol extracts of the selected marine samples.

Group	Conc. (µg/mL)	% of Denaturation Inhibition
Standard (Diclofenac)	1000	83.05
	500	74.58
	250	66.10
	125	54.24
	62.5	42.37
PA	1000	80.34
	500	63.73
	250	55.59
	125	44.07
	62.5	32.20
SPT	1000	76.95
	500	69.66
	250	57.63
	125	44.58
	62.5	33.73
HA	1000	81.35
	500	69.49
	250	59.32
	125	54.24
	62.5	40.68
HV	1000	80.17
	500	72.88
	250	64.07
	125	53.22
	62.5	43.39

PA = *Padina australis*, SPT = *Spatoglossum asperum*, HA = *Holothuria atra*, HV = *Hypnea valentiae*. This assay was conducted as a preliminary screening, and no inferential statistical analysis was applied.

**Table 9:** Drug-likeness Study of Phytochemicals of *Holothuria atra*, *Hypnea valentiae*, *Padina australis*, and *Spatoglossum asperum*. MW = Molecular Weight, HBA = Hydrogen Bond Acceptor, HBD = Hydrogen Bond Donor, nRB = Number of Rotational Bonds, TPSA = Topological Polar Surface Area, AMT = Ames Toxicity, AOT = Acute Oral Toxicity, HIA = Human Intestinal Absorption, B.S. = Bioavailability Score, BBB = Blood Brain Barrier, NAT = Not Ames Toxic, C = Carcinogens, and NC = Not Carcinogenic.

Plant	Compound Name	Lipinski Rules				Lipinski’s Violation ≤ 1	Veber’s Rules		Toxicity Parameters					
		MW (g/mol) <500	HBA <10	HBD <5	Log *p* ≤ 5		n RB ≤10	TPSA ≤ 140 (Å^2^)	AMT	CAR	AOT	HIA	B.S.	BBB
*Holothuria atra*	Propylhexedrine	155.28	1	1	2.5647	0	3	12.03	NAT	NC	II	0.9657	0.55	0.9829
	Trans-caryophyllene	204.35	0	0	4.7252	1	0	0	NAT	NC	III	0.9926	0.55	0.9536
	Methyl arachidonate	318.49	2	0	6.3051	-	15	26.3	NAT	C	III	0.9941	-	0.9838
	Bupranolol	271.78	3	2	2.7762	0	6	41.49	NAT	NC	III	0.9784	0.55	0.7572
	Phendimetrazine	191.27	2	0	2.0782	0	1	12.47	NAT	NC	III	0.9969	0.55	0.9846
*Hypnea valentiae*	Phytol acetate	338.57	2	0	6.9349	1	15	26.3	NAT	C	III	0.9937	0.55	0.9398
	3,7,11,15-Tetramethyl-2-hexadecen-1-ol	296.53	1	1	6.3641	1	13	20.23	NAT	NC	III	0.9846	0.55	0.9375
	Hexadecanoic acid	256.42	2	1	5.5523	1	14	37.3	NAT	NC	IV	0.9888	0.85	0.9488
	Retinoic acid, methyl ester	314.46	2	0	5.691	-	6	26.3	NAT	NC	III	0.9945	-	0.9307
	Lucenin 2	610.52	16	12	−2.688	3	5	291.43	AT	NC	IV	0.9156	0.17	0.6871
*Padina australis*	Pentadecanoic acid, 14-methyl ester	270.45	2	0	5.4966	1	14	26.3	NAT	C	III	0.976	0.55	0.9791
	1,2-Benzenedicarboxylic acid, butyl octyl ester	334.45	4	0	5.1608	1	14	52.6	NAT	NC	IV	0.977	0.55	0.9455
	10-Octadecenoic acid, methyl ester	310.47	3	0	5.3759	0	16	43.37	NAT	NC	III	0.9923	0.55	0.971
	Octadecenoic acid, methyl ester	296.49	2	0	6.1969	1	16	26.3	NAT	C	III	0.9952	0.55	0.9818
	Oleic acid	282.46	2	1	6.1085	1	15	37.3	NAT	NC	IV	0.9945	0.85	0.9539
*Spatoglossum asperum*	Palmitic acid methyl ester	270.45	2	0	5.6407	1	15	26.3	NAT	C	III	0.9881	0.55	0.9848
	Phytol	296.53	1	1	6.3641	1	13	20.23	NAT	NC	III	0.9846	0.55	0.9375
	Retinol acetate	328.49	2	0	6.0811	-	7	26.3	NAT	NC	III	0.9953	-	0.966
	Benzophenone	182.22	1	0	2.9176	0	2	17.07	NAT	NC	IV	0.9974	0.55	0.9841
	Fucosterol	412.69	1	1	7.9449	1	5	20.23	NAT	NC	I	1	0.55	0.9749

**Table 10:** Docking Scores of Selected Phytoconstituents of *Holothuria atra*, *Hypnea valentiae*, *Padina australis*, and *Spatoglossum asperum* for analgesic, anti-inflammatory, antioxidant, and antiarthritic activity.

Plant	Compounds	PubChem ID	Docking Score (Kcal/mol)			
			Analgesic (6cox)	Anti-Inflammatory (6y3c)	Antioxidant (1xan)	Antiarthritic (2az5)
*Holothuria atra*	Propylhexedrine	7558	−6.1	−5.2	−4.6	−4.6
	Trans-caryophyllene	5281515	−5.8	−6.2	−6.6	−5.4
	Methyl arachidonate	6421258	−7.5	−5.1	−5.7	−4.2
	Bupranolol	2475	−6.7	−5.8	−4.9	−5.6
	Phendimetrazine	30487	−6.9	−5.6	−5.5	−4.9
*Hypnea* *valentiae*	Phytol acetate	6428538	−7.9	−5.5	−5.7	−4.6
	3,7,11,15-Tetramethyl-2-hexadecen-1-ol	5366244	−7.1	−5.1	−5.4	−4.3
	Hexadecanoic acid	985	−6.6	−4.8	−4.7	−4.2
	Retinoic acid, methyl ester	5378821	−7.2	−6.5	−7.2	−5.9
	Lucenin 2	44257937	−7.4	−8.5	−7.1	−6.1
*Padina* *australis*	Pentadecanoic acid, 14-methyl ester	21205	−6.6	−4.8	−5.1	−4.3
	1,2-Benzenedicarboxylic acid, butyl octyl ester	66540	−7	−4.8	−5	−4.2
	10-Octadecenoic acid, methyl ester	6386109	−6.7	−4.6	−5.4	−4.3
	Octadecenoic acid, methyl ester	5370350	−6.3	−5.1	−5.2	−3.8
	Oleic acid	445639	−7	−5.8	−4.7	−4.3
*Spatoglossum asperum*	Palmitic acid methyl ester	8181	−5.8	−6.5	−5.9	−5.6
	Phytol	5280435	−7.3	−5.1	−4.8	−4.4
	Retinol acetate	638034	−7.5	−6.7	−7.2	−6.1
	Benzophenone	3102	**−8**	−7.1	−6.4	−5.7
	Fucosterol	5281328	−7.2	−8.2	−7.8	−7.1
	Standards (Diclofenac/Diclofenac/Ascorbic acid/Diclofenac)		−8.4	−6.4	−7.3	−5.8

**Table 11:** Binding Affinity and Non-binding Interactions of the Selected Phytochemicals of *Holothuria atra* (HA), *Hypnea valentiae* (HV), *Padina australis* (PA), and *Spatoglossum asperum* (SA) for Analgesic (6cox), Anti-inflammatory (6y3c), Antioxidant (1xan), and Antiarthritic (2az5) Activity.

Section Number	Receptor	Compounds Name	Binding Affinity (kcal/mol)	Bond Type	Amino Acids
1	6cox	Benzophenone (SA)	−8	Conventional Hydrogen Bond	SER530
				Pi-Sulfur	MET522
				Pi-Pi T-shaped	TRP387
				Pi-Alkyl	VAL349, ALA527, LEU531
		Phytol acetate (HV)	−7.9	Conventional Hydrogen Bond	PHE518
				Alkyl	VAL349 (3), VAL523, ALA527 (2), LEU384, LEU352 (2), LEU531 (2), VAL116, LEU359
				Pi-Alkyl	TYR355, TYR385, TRP387 (2), PHE518
		Methyl arachidonate (HA)	−7.5	Carbon Hydrogen Bond	LEU352
				Alkyl	VAL349 (3), ALA516, VAL523 (3), ALA527, MET522, LEU531, LEU352
				Pi-Alkyl	HIS90, TYR355, TYR385, TRP387, PHE518 (3)
		Diclofenac (Standard)	−8.4	Pi-Pi T-shaped	TRP387
				Pi-Alkyl	VAL349, VAL523, ALA527, LEU352
2	6y3c	Lucenin 2 (HV)	−8.5	Conventional Hydrogen Bond	THR89, ARG120, GLU524 (2), PRO84
				Carbon Hydrogen Bond	ARG79, ASN80, PRO86
				Pi-Alkyl	ARG83 (2), PRO84
		Fucosterol (SA)	−8.2	Alkyl	VAL119 (2), LEU123, ARG79
				Pi-Alkyl	HIS43, TYR64
		Benzophenone (SA)	−7.1	Carbon Hydrogen Bond	SER353 (2)
				Pi-Cation	ARG120
				Pi-Pi Stacked	PHE518
				Pi-Alkyl	LEU352, ALA527 (2), VAL349, LEU531
		Diclofenac (Standard)	−6.4	Conventional Hydrogen Bond	ARG120
				Pi-Alkyl	LEU115, VAL116, VAL119, LEU93, LEU112
3	1xan	Fucosterol (SA)	−7.8	Pi-Sigma	PHE78
				Alkyl	VAL74 (2)
				Pi-Alkyl	HIS75, PHE78 (2), HIS82, PHE87 (2), TYR407
		Retinoic acid, methyl ester (HV)	−7.2	Conventional Hydrogen Bond	SER470
				Carbon Hydrogen Bond	LEU438
				Alkyl	LEU438
				Pi-Alkyl	PHE78 (4), HIS82, TYR85 (2), TYR407 (2)
		Retinol acetate (SA)	−7.2	Conventional Hydrogen Bond	GLY439
				Carbon Hydrogen Bond	LEU438
				Pi-Sigma	TYR85
				Alkyl	LEU438
				Pi-Alkyl	PHE78 (4), HIS82 (2), TYR85, TYR407 (2)
		Ascorbic acid (Standard)	−7.3	Conventional Hydrogen Bond	TYR85, ASP441
				Carbon Hydrogen Bond	PRO375, PRO376, TYR407
				Pi-Sigma	LEU438
				Pi-Pi T-shaped	TYR407
4	2az5	Fucosterol (SA)	−7.1	Carbon Hydrogen Bond	LEU120
				Pi-Sigma	TYR59
				Alkyl	LEU36, VAL13
				Pi-Alkyl	HIS15 (2), TYR59 (2), TYR151
		Lucenin 2 (HV)	−6.1	Conventional Hydrogen Bond	LEU120
				Carbon Hydrogen Bond	GLY121, GLN61
				Pi-Pi Stacked	TYR59
		Retinol acetate (SA)	−6.1	Conventional Hydrogen Bond	TYR119
				Pi-Alkyl	TYR59 (3), TYR119 (3), TYR151
		Diclofenac (Standard)	−5.8	Conventional Hydrogen Bond	SER60, LEU120
				Pi-Pi Stacked	TYR59
				Pi-Alkyl	HIS15, TYR59, TYR151

**Table 12:** PASS Prediction of the Reported Biologically Active Compounds of *Holothuria atra*, *Hypnea valentiae*, *Padina australis*, and *Spatoglossum asperum*.

Plant	Compound Name	Biological Activity							
		Analgesic		Anti-Inflammatory		Antioxidant		Antiarthritic	
		Pa	Pi	Pa	Pi	Pa	Pi	Pa	Pi
*Holothuria atra*	Propylhexedrine	0.508	0.032	0.271	0.130	-	-	0.253	0.173
	Trans-caryophyllene	0.414	0.099	0.745	0.011	0.174	0.075	0.482	0.058
	Methyl arachidonate	0.593	0.008	0.728	0.013	0.296	0.024	0.484	0.058
	Bupranolol	0.250	0.099	0.304	0.073	0.140	0.115	0.256	0.171
	Phendimetrazine	0.477	0.051	-	-	-	-	0.311	0.130
*Hypnea valentiae*	Phytol acetate	0.242	0.112	0.600	0.032	0.480	0.007	0.281	0.151
	3,7,11,15-Tetramethyl-2-hexadecen-1-ol	0.300	0.182	0.458	0.070	0.475	0.008	0.387	0.091
	Hexadecanoic acid	0.526	0.023	0.515	0.052	0.222	0.045	0.528	0.046
	Retinoic acid, methyl ester	-	-	0.653	0.022	0.673	0.004	0.329	0.119
	Lucenin 2	0.324	0.165	0.533	0.047	0.847	0.003	0.382	0.093
*Padina australis*	Pentadecanoic acid, 14-methyl ester	0.490	0.042	0.392	0.100	0.243	0.038	0.479	0.059
	1,2-Benzenedicarboxylic acid, butyl octyl ester	0.453	0.068	0.503	0.056	0.151	0.101	0.470	0.062
	10-Octadecenoic acid, methyl ester	0.487	0.044	0.428	0.082	0.259	0.033	0.465	0.063
	Octadecenoic acid, methyl ester	0.553	0.015	0.563	0.040	0.340	0.018	0.520	0.048
	Oleic acid	0.561	0.013	0.614	0.029	0.283	0.026	0.501	0.053
*Spatoglossum asperum*	Palmitic acid methyl ester	0.538	0.019	0.510	0.054	0.210	0.050	0.548	0.042
	Phytol	0.300	0.182	0.458	0.070	0.475	0.008	0.387	0.091
	Retinol acetate	-	-	0.726	0.013	0.652	0.004	0.227	0.197
	Benzophenone	0.501	0.036	0.703	0.015	0.171	0.078	0.801	0.008
	Fucosterol	0.540	0.018	0.575	0.037	0.196	0.057	0.490	0.056

## Data Availability

All data supporting the findings of this study are included within the article and its Supplementary Materials.

## Supplementary Materials

The following supporting information can be downloaded at: https://www.mdpi.com/article/10.3390/bioengineering13020158/s1, Table S1: Individual mice weights at different time intervals for acute toxicity evaluation; Table S2: Writhing count of mice to evaluate peripheral analgesic activity by acetic acid induced writhing method; Table S3: Reaction time of individual mice at different time intervals to evaluate central analgesic activity by tail immersion method; Table S4: Measurement of individual paw circumference of mice to evaluate anti-inflammatory activity; Table S5: Absorbances at different concentrations of the standard and the crude ethanol extract of the selected samples by DPPH assay; Table S6: Measurement of absorbance at different concentrations of the marine samples and the standard to evaluate anti-arthritic activity.

## Abbreviations

The following abbreviations are used in this manuscript: SPT = Ethanol extract of *Spatoglossum asperum*; PA = Ethanol extract of *Padina australis*; HA = Ethanol extract of *Holothuria (Halodeima) atra*; HV = Ethanol extract of *Hypnea valentiae*.
