# Supplementary material for: Integrated In Vitro, In Vivo, and In Silico Evaluation of Antioxidant, Anti-Inflammatory, Analgesic, and Anti-Arthritic Activities of Selected Marine Species
Source: Bioengineering (Basel). 2026 Jan 28;13(2):158. doi: 10.3390/bioengineering13020158 (PMC12938147; doi:10.3390/bioengineering13020158)
Supplement: Supplementary file 1 [file bioengineering-13-00158-s001.zip › bioengineering-4043341-supplementary.pdf]

Supplementary File

Integrated In Vitro, In Vivo, and In Silico Evaluation of Antioxidant, Anti-Inflammatory, Analgesic, and Anti-Arthritic Activities of Selected Marine Species

Md. Jahin Khandakar [1], Ainun Nahar [2,3], Md. Wahidul Alam [1], Md. Jahirul Islam Mamun [3], Abu Sayeed Muhammad Sharif [4], Asef Raj [5], Md. Enamul Hoque [1], Israt Sultana Isha [1], Nafisa Nawsheen [3], Saika Ahmed [6], Md Riasat Hasan [7], Abu Bin Ihsan [2,*] and Takashi Saito [7,*]

[1] Department of Oceanography, Faculty of Marine Sciences and Fisheries, University of Chittagong, Chittagong 4331, Bangladesh; khandakarjahin@gmail.com (M.J.K.); wahidul.alam@cu.ac.bd (M.W.A.); enamul_imsf@cu.ac.bd (M.E.H.); isratisha055@gmail.com (I.S.I.)
[2] Department of Pharmacy, School of Life Sciences, Eastern University, Dhaka 1345, Bangladesh; tahira.ainun@gmail.com
[3] Department of Pharmacy, Faculty of Biological Sciences, University of Chittagong, Chittagong 4331, Bangladesh; jahirulmamun3@gmail.com (M.J.I.M.); nafisanawsheen333@gmail.com (N.N.)
[4] Department of Biological Oceanography, Bangladesh Oceanographic Research Institute, Cox's Bazar, Chittagong 4750, Bangladesh; sharif.asm@bori.gov.bd
[5] School of Pharmacy, BRAC University, Dhaka 1212, Bangladesh; raj.asef@gmail.com
[6] Department of Chemistry, University of Dhaka, Dhaka 1000, Bangladesh; saika@du.ac.bd
[7] Division of Clinical Cariology and Endodontology, Department of Oral Rehabilitation, School of Dentistry, Health Sciences University of Hokkaido, Tobetsu 061-0293, Hokkaido, Japan; riasat@hoku-iryo-u.ac.jp
* Correspondence: ihsan.pharmacy@easternuni.edu.bd (A.B.I.); t-saito@hoku-iryo-u.ac.jp (T.S.)

Table of Contents

Table S1: Individual mice weights at different time intervals for acute toxicity evaluation.

Table S2. Writhing count of mice for evaluation of peripheral analgesic activity using the acetic acid–induced writhing method.

Table S3. Reaction time (latency) of individual mice at different time intervals for evaluation of central analgesic activity using the tail immersion method.

Table S4. Measurement of individual paw circumference of mice for evaluation of anti-inflammatory activity.

Table S5. Absorbance values at different concentrations of the standard and marine ethanol extracts assessed by DPPH radical scavenging assay.

Table S6. Absorbance measurements at different concentrations of marine samples and standard for evaluation of anti-arthritic activity (protein denaturation assay).

Table S1: Individual mice weights at different time intervals for acute toxicity evaluation.

Mouse ID	Day 0 (g)	Day 7 (g)	Day 14 (g)
**Control (1% Carboxymethyl cellulose gel)**			
M1	25.0	25.4	25.9
M2	25.6	26.0	26.3
M3	26.0	26.5	26.9
M4	26.4	27.0	27.4
M5	27.0	27.6	27.9
Hypnea valentiae			
M1	25.8	26.4	26.9
M2	26.1	26.7	27.2
M3	26.5	26.9	27.3
M4	25.9	26.3	26.8
M5	26.2	26.8	27.1
Padina australis			
M1	25.3	25.8	26.3
M2	25.5	26.2	26.7
M3	25.9	26.4	26.8
M4	26.3	26.7	27.2
M5	26.5	26.9	27.3
Spatoglossum asperum			
M1	25.6	26.0	26.4
M2	25.8	26.4	26.8
M3	26.4	26.7	27.0
M4	26.5	27.2	27.5
M5	26.9	27.3	27.6
Holothuria atra			
M1	25.2	25.5	26.3
M2	25.5	26.0	26.4
M3	25.8	26.3	26.6
M4	26.1	26.5	26.9
M5	26.4	26.6	27.0

Table S2: Writhing count of mice to evaluate peripheral analgesic activity by acetic acid induced writhing method.

Animal Group	Writhing count							% of Writhing
	M-1	M-2	M-3	M-4	M-5	Average	Total	
Control	45.5	46	48	47	48	46.90	234.5	100
Standard	7	5	6	5	9	6.40	32	13.65
PA-400	14	12	13	17	16	14.40	72	30.70
PA-200	17	15	15	19	18	16.80	84	35.82
SPT-400	14	12	14	13	14	13.40	67	28.57
SPT-200	24	26	28	25	24	25.40	127	54.15
HA-400	13	11	16	12	11	12.60	63	26.86
HA-200	24	23	21	25	22	23.00	115	49.04
HV-400	8	9	11	7	6	8.20	41	17.48
HV-200	17	19	15	18	16	17.00	85	36.25

Table S3: Reaction time of individual mice at different time intervals to evaluate central analgesic activity by tail immersion method.

Group	No. of mice	Pre-treatment	30min	60min	90min	120min
1.Control	1	0.50	1.30	1.50	0.98	1.20
	2	0.69	0.75	1.10	1.87	0.50
	3	0.40	1.00	1.30	1.19	0.98
	4	0.60	0.64	0.89	1.25	1.20
	5	0.56	1.30	1.00	1.12	1.10
2.Standard	1	3.25	5.89	5.26	5.19	4.87
	2	3.10	10.07	11.09	10.03	9.86
	3	2.89	10.18	11.11	10.23	10.21
	4	3.57	12.03	11.62	9.97	8.85
	5	2.67	7.09	10.11	11.03	11.87
3.PA-400	1	1.22	1.48	0.98	4.75	1.98
	2	1.23	1.67	1.30	4.58	1.67
	3	1.01	1.54	1.20	5.89	1.72
	4	1.20	1.23	1.50	4.78	1.98
	5	1.30	1.43	1.40	5.78	1.12
4.PA-200	1	1.30	1.64	3.10	3.40	1.07
	2	1.40	1.98	2.40	3.80	1.80
	3	1.20	1.43	4.80	4.60	1.50
	4	1.00	1.45	3.40	3.20	1.36
	5	1.10	1.56	3.60	3.10	1.60
5.HV-400	1	1.40	1.61	0.81	1.21	1.26
	2	1.20	1.73	1.20	1.48	1.36
	3	1.10	1.54	1.30	1.98	2.42
	4	1.00	1.89	1.50	2.10	1.01
	5	1.30	1.38	1.50	1.10	1.00
6.HV-200	1	1.08	1.31	1.50	1.18	1.50
	2	1.01	1.29	1.90	1.24	1.90
	3	1.23	1.47	1.30	1.64	2.20
	4	1.11	1.34	1.70	1.89	1.40
	5	1.09	1.31	1.30	1.11	1.20
7.SPT-400	1	0.53	1.10	1.41	1.38	1.61
	2	0.67	1.20	1.28	1.56	1.85
	3	0.75	1.20	1.37	1.78	1.56
	4	0.48	1.30	1.54	1.28	1.78
	5	0.43	1.10	1.41	1.43	1.00
8.SPT-200	1	0.83	1.10	1.49	1.36	0.81
	2	0.99	1.20	1.56	1.12	0.90
	3	0.78	1.30	1.38	1.48	2.10
	4	0.69	1.40	1.44	1.56	1.68
	5	0.96	1.40	1.48	1.19	1.31

9.HA-400	1	0.61	1.41	1.10	1.92	1.19
	2	0.59	1.24	1.30	1.67	2.30
	3	0.76	1.38	1.50	1.89	2.50
	4	0.49	1.12	1.50	2.34	2.20
	5	0.66	1.20	1.60	1.95	1.84
10.HA-200	1	0.71	1.06	1.36	1.20	1.92
	2	0.47	1.10	1.38	1.30	1.72
	3	0.80	1.00	1.32	1.40	1.56
	4	1.28	1.20	1.24	1.50	2.38
	5	1.34	0.99	1.48	1.60	3.42

Reaction time and baseline latency were recorded in seconds (s) using a smartphone stopwatch with a display resolution of 0.01 s.

Table S4: Measurement of individual paw circumference of mice to evaluate anti-inflammatory activity.

Group	Mice no.	Pre-injection paw circumference (cm)	Post-injection paw circumference (cm)			
		0 hour	1st hour	2nd hour	3rd hour	4th hour
1.Ctrl	1	1.6	2	2	2	2
	2	1.6	2.2	2.2	2.1	2
	3	1.3	1.6	1.6	1.6	1.6
	4	1.2	1.6	1.6	1.5	1.5
	5	1.1	1.7	1.7	1.7	1.6
2.Std	1	1.2	1.3	1.2	1.2	1.2
	2	1.2	1.2	1.2	1.2	1.2
	3	1.3	1.4	1.4	1.3	1.3
	4	1.2	1.3	1.3	1.2	1.2
	5	1.1	1.1	1.1	1.1	1.1
3.PA -400	1	1.2	1.5	1.4	1.3	1.2
	2	1.2	1.6	1.4	1.2	1.2
	3	1	1.4	1.3	1.1	1
	4	1.3	1.6	1.5	1.4	1.3
	5	1.3	1.5	1.3	1.3	1.3
4.PA -200	1	1.3	1.8	1.4	1.3	1.3
	2	1.2	1.9	1.6	1.3	1.2
	3	1.2	1.7	1.3	1.2	1.2
	4	1.2	1.9	1.5	1.4	1.3
	5	1.1	1.8	1.4	1.2	1.1
5.HV -400	1	1.1	1.2	1.1	1.1	1.1
	2	1.2	1.4	1.2	1.2	1.2
	3	1.1	1.3	1.1	1.1	1.1
	4	1.2	1.2	1.2	1.2	1.2
	5	1.4	1.5	1.4	1.4	1.4

6.HV -200	1	1.4	1.5	1.5	1.4	1.4
	2	1.5	1.6	1.5	1.5	1.5
	3	1.5	1.6	1.3	1.4	1.5
	4	1.3	1.6	1.5	1.4	1.3
	5	1.5	1.8	1.6	1.5	1.5
7.SPT -400	1	1.2	1.4	1.3	1.3	1.2
	2	1.2	1.4	1.4	1.3	1.2
	3	1.2	1.5	1.3	1.2	1.2
	4	1.3	1.6	1.4	1.3	1.3
	5	1.4	1.7	1.6	1.5	1.4
8.SPT -200	1	1.3	1.4	1.4	1.3	1.3
	2	1.4	1.6	1.5	1.4	1.4
	3	1.2	1.6	1.4	1.2	1.2
	4	1.3	1.6	1.4	1.3	1.3
	5	1.2	1.5	1.3	1.2	1.2
9.HA -400	1	1.4	1.5	1.4	1.4	1.4
	2	1.6	1.7	1.6	1.6	1.6
	3	1.5	1.7	1.5	1.5	1.5
	4	1.3	1.4	1.3	1.3	1.3
	5	1.3	1.4	1.3	1.3	1.3
10.HA -200	1	1.3	1.5	1.4	1.3	1.3
	2	1.2	1.4	1.3	1.2	1.2
	3	1.1	1.2	1.1	1.1	1.1
	4	1	1.1	1	1	1
	5	1.2	1.4	1.2	1.2	1.2

Table S5: Absorbances at different concentrations of the standard and the crude ethanol extract of the selected samples by DPPH assay.

Group	Concentration(µg/ml)	LogC	Absorbance
Negative control			0.311
Standard	500	2.69897	0.4498
	250	2.39794	0.4769
	125	2.09691	0.4874
	62.5	1.79588	0.7462
	31.25	1.49485	0.7947
	15.63	1.19395	0.8245
SPT	500	2.69897	0.0706
	250	2.39794	0.1797
	125	2.09691	0.2959
	62.5	1.79588	0.3577
	31.25	1.49485	0.5053
	15.63	1.19395	0.5502
HV	500	2.69897	0.1694
	250	2.39794	0.2063
	125	2.09691	0.2861

	62.5	1.79588	0.4736
	31.25	1.49485	0.5531
	15.63	1.19395	0.5618
PA	500	2.69897	0.017
	250	2.39794	0.019
	125	2.09691	0.023
	62.5	1.79588	0.062
	31.25	1.49485	0.063
	15.63	1.19395	0.059
HA	500	2.69897	0.024
	250	2.39794	0.025
	125	2.09691	0.026
	62.5	1.79588	0.064
	31.25	1.49485	0.065
	15.63	1.193959	0.066

Table S6: Measurement of absorbance at different concentrations of the marine samples and the standard to evaluate anti-arthritic activity

Group	Concentration (µg/ml)	Absorbance of test sample	Product control absorbance	Abs. of test control	% of denaturation inhibition
Standard	1000	0.023	0.013	0.059	83.05
	500	0.026	0.011		74.58
	250	0.029	0.009		66.10
	125	0.033	0.006		54.24
	62.5	0.037	0.003		42.37
PA	1000	0.031	0.0194	0.059	80.34
	500	0.039	0.0176		63.73
	250	0.041	0.0148		55.59
	125	0.047	0.014		44.07
	62.5	0.051	0.011		32.20
SPT	1000	0.046	0.0324	0.059	76.95
	500	0.049	0.0311		69.66
	250	0.054	0.029		57.63
	125	0.057	0.0243		44.58
	62.5	0.059	0.0199		33.73
HA	1000	0.022	0.011	0.059	81.35
	500	0.025	0.007		69.49
	250	0.029	0.005		59.32
	125	0.031	0.004		54.24
	62.5	0.037	0.002		40.68
HV	1000	0.0197	0.008	0.059	80.17
	500	0.022	0.006		72.88

	250	0.0263	0.0051		64.07
	125	0.031	0.0034		53.22
	62.5	0.036	0.0026		43.39

Note: SPT = Ethanol extract of *Spatoglossum asperum;* PA = Ethanol extract of *Padina australis;* HA= Ethanol extract of *Holothuria (Halodeima) atra;* HV = Ethanol extract of *Hypnea valentiae.*
